# Engineering Archeal Surrogate Systems for the Development of Protein–Protein Interaction Inhibitors against Human RAD51

**DOI:** 10.1016/j.jmb.2016.10.009

**Published:** 2016-11-20

**Authors:** Tommaso Moschetti, Timothy Sharpe, Gerhard Fischer, May E. Marsh, Hong Kin Ng, Matthew Morgan, Duncan E. Scott, Tom L. Blundell, Ashok R. Venkitaraman, John Skidmore, Chris Abell, Marko Hyvönen

**Affiliations:** 1 Department of Biochemistry, University of Cambridge, 80 Tennis Court Road, Cambridge, CB2 1GA, UK; 2 Department of Chemistry, University of Cambridge, Lensfield Road, Cambridge CB2 1EW, UK; 3 Medical Research Council Cancer Unit, University of Cambridge, Hills Road, Cambridge CB2 0XZ, UK

**Keywords:** PPI, protein–protein interaction, HR, homologous recombination, NF, nucleoprotein filament, BRC4, fourth BRC repeat, HumRadA1, first humanised RadA protein, DSF, differential scanning fluorimetry, ITC, isothermal titration calorimetry, FP, fluorescence polarisation, GST, glutathione *S*-transferase, DLS, dynamic light scattering, PEG, polyethylene glycol, TEV, tobacco etch mosaic virus, DMSO, dimethyl sulphoxide, recombinase, protein engineering, humanisation, surrogate system, fragment-based drug discovery

## Abstract

Protein–protein interactions (PPIs) are increasingly important targets for drug discovery. Efficient fragment-based drug discovery approaches to tackle PPIs are often stymied by difficulties in the production of stable, unliganded target proteins. Here, we report an approach that exploits protein engineering to “humanise” thermophilic archeal surrogate proteins as targets for small-molecule inhibitor discovery and to exemplify this approach in the development of inhibitors against the PPI between the recombinase RAD51 and tumour suppressor BRCA2. As human RAD51 has proved impossible to produce in a form that is compatible with the requirements of fragment-based drug discovery, we have developed a surrogate protein system using RadA from *Pyrococcus furiosus.* Using a monomerised RadA as our starting point, we have adopted two parallel and mutually instructive approaches to mimic the human enzyme: firstly by mutating RadA to increase sequence identity with RAD51 in the BRC repeat binding sites, and secondly by generating a chimeric archaeal human protein. Both approaches generate proteins that interact with a fourth BRC repeat with affinity and stoichiometry comparable to human RAD51. Stepwise humanisation has also allowed us to elucidate the determinants of RAD51 binding to BRC repeats and the contributions of key interacting residues to this interaction. These surrogate proteins have enabled the development of biochemical and biophysical assays in our ongoing fragment-based small-molecule inhibitor programme and they have allowed us to determine hundreds of liganded structures in support of our structure-guided design process, demonstrating the feasibility and advantages of using archeal surrogates to overcome difficulties in handling human proteins.

## Highlights

• Archeal RadA, humanised to resemble RAD51, binds the fourth BRC repeat with high affinity.
• Chimeric archeal/human RAD51 shows increased thermal stability.
• Robust crystallographic system enables fragment-based drug discovery against RAD51.
• Dedicated surrogate proteins were needed for different assays.

## Graphical Abstract

**Figure:**
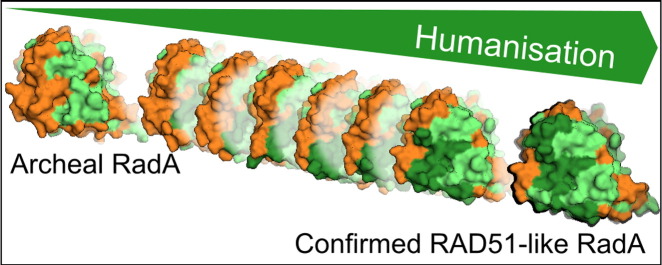

## Introduction

Currently available drugs are mainly active against a subset of “druggable” protein domains. Such bias limits the development of new therapies and leaves a pressing need for the identification of novel targets [1]. Protein–protein interaction (PPI) interfaces represent a class of binding sites that play key roles in all biological processes, accounting for approximately 130,000 binary interactions in human [2]. These are regarded as a huge reservoir of potential new targets by modern pharmacology, provided that specific limitations can be overcome; as opposed to enzyme active sites, PPI interfaces are large, almost featureless, and generally have not evolved to bind small ligands [3]. They may thus appear unsuitable as drug targets at first glance [4], [5]. Nevertheless, the advent of a combination of structural biology methods and extensive computational analyses has helped to develop potent PPI binders, a number of which have reached clinical phase trials during the last decade [6].

Successful development of PPI inhibitors also implicates the possibility to inhibit the activity of multiprotein complexes regulating DNA repair, cell division, and other fundamental cellular processes. Although these macromolecular machineries are attractive drug targets, their complexity can be seen as a practical limitation for the development of inhibitors. In this respect, our purpose was to demonstrate that fragment-based drug discovery methods could be successfully used to target those PPIs that lie at the core of large regulatory multiprotein complexes. We focused our attention on human RAD51, an ATP-dependent recombinase crucial for homologous recombination (HR) [7] and for error-free repair of DNA double-strand breaks. RAD51 is also required in normal cells to enable correct cell division; RAD51 deletion is embryonic lethal in vertebrates, whereas its conditional knockout leads to rapid mitotic failure due to chromosomal aberrations [8], [9]. Inhibitors against RAD51 could be used as therapeutic agents to treat cancer, either alone or in conjunction with DNA-damaging agents or ionising radiation.

Together with archeal RadA and bacterial RecA, RAD51 forms a larger class of recombinases, which share many structural and functional characteristics [10], most notably the ability to oligomerise on DNA substrates to form ordered nucleoprotein filaments (NFs). NFs catalyse the reactions leading to the homologous strand pairing and strand-exchange that underlie HR. In human cells, the correct execution of RAD51-dependent HR requires the concerted effort of several accessory proteins, including the BRCA2 tumour suppressor. The inheritance of germline mutations affecting BRCA2 predisposes to breast, ovary, pancreas, and prostate cancers (reviewed in Ref. [11]). BRCA2 binds directly to RAD51 through eight ~ 35-residue-long motifs, the BRC repeats, whose sequence and spacing are evolutionarily conserved within a large ~ 1000-residue region of BRCA2. BRCA2-deficient cells fail to form nuclear RAD51 foci at the sites of DNA damage [12], [13], due to the loss of essential functions of BRCA2 in regulating RAD51 dynamics [14] and nuclear–cytoplasmic transport [15]. *In vitro*, RAD51-dependent HR reactions under physiologic ionic conditions are promoted by the BRC repeats [16], which work to enhance the nucleation and stability of RAD51 assembly on single-stranded DNA whilst inhibiting the premature formation of RAD51:double-stranded DNA assemblies [17], [18], [19], [20], [21]. BRCA2-deficient cells exhibit increased sensitivity to radiation and chemical DNA-damaging agents [13], [22], [23]. Moreover, there is evidence that RAD51 is overexpressed in certain cancer cells, where expression levels correlate with the aggressiveness of the tumour and its resistance to chemotherapy [24]. These findings suggest that small molecules targeting the BRCA2–RAD51 interaction may be useful in new approaches for cancer therapy.

**Fig. 1:**
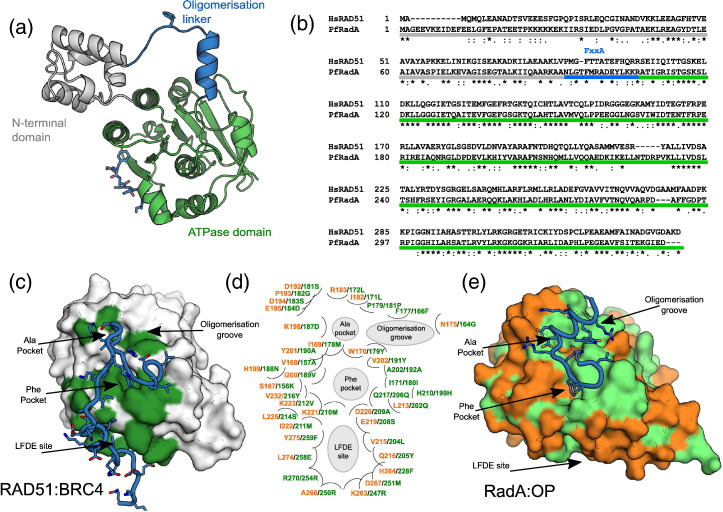
Comparative analysis of *Hs*RAD51 and *Pf*RadA. (a) Domain structure of RAD51 with N-terminal domain in grey, FxxA containing linker in blue, and ATPase domain in green. (b) Alignment of human RAD51 and *P. furiosus* RadA, with different domains highlighted in the same colours as the structure in panel (a). Asterisks indicate identical residues between the two proteins. (c–e) Comparison of conservation between RAD51 and RadA in and around the BRC4 binding site in RAD51. (c) RAD51 (surface representation) in complex with BRC4 peptide (blue tube with side chains as sticks; PDB: 1N0W) shows the BRC4 interacting residues in green on the surface. (d) Schematic map of the residues in the extended BRC4 binding site and oligomerisation groove, with RadA residues labelled in green and orange for identical or non-identical residues with RAD51, respectively, followed by RAD51 residue labels in green. Different parts of the BRC repeat and oligomerisation epitope binding sites are highlighted in grey. For orientation, the positions of the labelled binding sites are approximately in the equivalent positions in the two proteins at either side. (e) Structure of RadA ATP domain (PDB: 1PZN, chain A) bound to the oligomerisation peptide (blue tube with side chains as sticks,). The surface of RadA ATPase domain is coloured light green for identical residues with RAD51 and orange for non-identical residues. The structures of (c) RAD51 and (e) RadA are shown in the same orientation after superpositioning.

The overall structural architecture is conserved between the eukaryotic RAD51 and archeal RadA [25], [26], [27], [28]. The recombinase is composed of two globular domains: an N-terminal helical domain that facilitates DNA binding and an AAA + family nucleotide-binding domain with ATPase activity (Fig. 1a). These are joined by a linker carrying a conserved FxxA epitope that drives self-association [26], [27], [29]. The conserved Phe and Ala residues of the FxxA motif bind in two small pockets on the ATPase domain, across the central β-sheet [30]. Downstream of the alanine, the linker folds into a short helix and establishes further interactions with an aromatic residue (Tyr or Phe; Fig. 1c) in a shallow pocket of the catalytic domain (the oligomerisation groove) [26].

The structure of RAD51 in complex with the fourth BRC repeat (BRC4) of BRCA2 provided a mechanistic explanation for the control exerted by BRCA2. The BRC repeat adheres to RAD51 in a way similar to a “Velcro” strip: through a large number of independent contacts over a wide surface. In analogy to the RAD51 oligomerisation linker, the N-terminus of BRC4 carries an FxxA motif that binds the RAD51 ATPase domain in the same FxxA pockets (see above). The C-terminal portion of BRC4 instead folds over the other side of RAD51, using a conserved LFDE motif to bind to what we call the LFDE pocket [25], [31]. *In vitro*, high concentrations of isolated BRC4 peptide are effective in disrupting RAD51 oligomers and NFs [32] and associate in cells with sensitivity to DNA-damaging agents [23].

Notably, RAD51 spontaneously folds into a globular architecture and generates an interface that, upon binding, induces the otherwise unstructured BRC repeat to assume the optimal binding conformation [33]. This is an example of a “concerted folding and binding” process that immediately suggested a strategy for a fragment-based drug discovery approach [34]: preventing the concerted folding–binding event through small molecules that target the interaction hotspots on the RAD51 interface. This seems preferable over targeting the whole complex with interfacial inhibitors that lock the two partners into a “dead-end binary complex” [35].

We pursued this strategy and developed small-molecule inhibitors against the BRCA2–RAD51 interaction using fragment-based and structure-guided approaches [36], [37]. These required extensive, iterative biophysical and crystallographic assessment of small molecules binding to guide the design of high-potency inhibitors. Therefore, the prerequisite for the success of the whole project was the availability of RAD51 as a stable and unliganded protein, and deprived from the ability to form oligomers that would prevent any fragment from binding to the target site. Such variant of RAD51 was not available at the beginning of the project and, despite extensive efforts, any attempt to generate it using the human protein failed [38]. We therefore explored the possibility of using a more stable orthologue as a surrogate and focused our efforts on archeal RadA from *Pyrococcus furiosus*, a protein that was already structurally characterised and is closely related to human RAD51 [26], [39]. All RAD51/RadA family recombinases self-associate through their N-terminal FxxA motifs to form helical oligomers. Such oligomeric structures are poorly suited for biophysical screening or structural studies aiming to characterise the interaction of potential inhibitors with the oligomerisation interface.

Here, we describe the complete process of generating robust, RAD51-like surrogate systems from this archeal protein *via* two distinct approaches: by stepwise mutation of the surface of the RadA to humanise the BRC4 binding area (HumRadA series of mutants) and by generating an archeal/human chimera (ChimRAD51) in which all of the BRC4 binding part of RAD51 is stabilised by parts of RadA. We present thorough structural and biophysical characterisation of the different surrogate proteins and demonstrate their suitability for structure-guided drug discovery, highlighting the potential of this approach for other hard-to-analyse targets.

## Results

### Humanisation of RadA

We have already reported the successful monomerisation of RadA previously, by removing the N-terminal the FxxA epitope that governs self-association, and shown that the C-terminal ATPase domain (RadA-ct) is correctly folded and able to bind ATP and short FxxA-like peptides [30], [38] (Fig. 1a). With a monomeric RadA in hand, the BRC4 binding site on RAD51 was analysed in more detail. The sequence identity between human RAD51 (*Hs*RAD51) and *P. furiosus* RadA (*Pf*RadA) in the C-terminal ATPase domain is only 53%. Although the two proteins self-associate with similar FxxA motifs (FHTA and FMRA, respectively; Fig. 1b), their FxxA binding sites are not identical in sequence and the LFDE sites are yet more divergent (Fig. 1c). As protein–ligand interactions can be greatly affected even by a single conservative mutation in the binding site, significant mutagenesis of surface residues was needed to create a viable surrogate system in which the target site would closely resemble the one of *Hs*RAD51.

We proceeded with the humanisation of RadA-ct in a stepwise fashion, starting from the immediate vicinity of the phenylalanine-binding pocket at the FxxA site (Phe pocket) and progressing towards the LFDE site (needed for high-affinity BRC4 binding) and the oligomerisation groove, always changing the RadA residues to corresponding human ones (Fig. 1c and Table 1). Initial analysis suggested that at least four residues surrounding the Phe pocket needed to be mutated: I169M_158_, Y201A_190_, and V202Y_191_, K221M_210_ (we will use RadA residue numbers to match with the residue numbering in our crystal structures, but indicate equivalent RAD51 residue numbers as a subscript). Mutation of these residues in the monomerised form of RadA was used to create the first humanised RadA protein (HumRadA1). This minimally humanised RadA mutant could be expressed and purified similar to the original monomerised protein; its crystal structure confirms the success of the mutagenesis and shows that the binding site resembles that of *Hs*RAD51, with the side chain of R204_194_ moving to the position found in RAD51 (Supplementary Table 1 and Supplementary Fig. 1). This mutant served as the first crystallographic model for the analysis of early fragment hits [37] and for the characterisation of FxxA-like tetrapeptide binding to RadA [30].

**Fig. 2:**
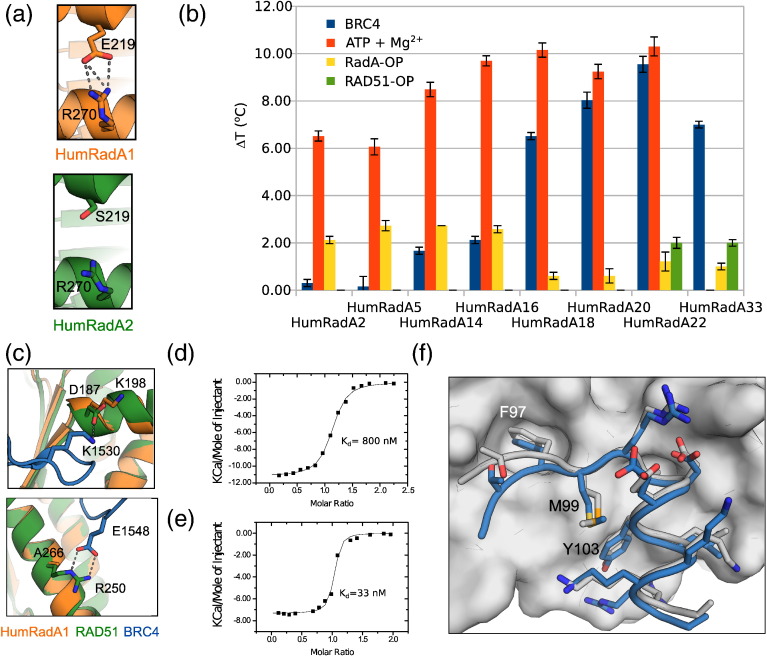
Humanisation of RadA and validation of mutants with different ligands. (a) Details of ionic interaction between E219 and R270 in HumRadA1 (orange) and the equivalent residues in HumRadA2 (green), causing reduction in the thermal stability of the protein. (b) Thermal shift analyses of different HumRadA mutants in the presence of BRC4 (blue), RadA-OP (yellow), and RAD51-OP (green) peptides and with ATP-Mg2 + (red). (c) Mutations A266R_250_ and K198D_187_ (introduced in HumRadA16 and HumRadA18, respectively) reinstate the interactions existing in the HsRAD51:BRC4 complex that promote the tight binding of the peptide. RAD51 structure is shown in green and RadA in orange, with BRC4 repeat in blue. RAD51 structure is shown in green and RadA in orange, with BRC4 repeat in blue. (d) Binding isotherm of ITC titration of RAD51 oligomerisation peptide into HumRadA33. (e) Binding isotherm of ITC titration of BRC4 peptide into HumRadA33. ITC data for other peptide binding ITC measurements are found in Fig. S3. (f) Structure of HumRadA1 in complex with RadA-OP peptide. The peptide (in blue) is shown as sticks on the HumRadA2 molecular surface. The white sticks show the corresponding region of the oligomerisation sequence from *Pf*RadA heptameric structure (PDB: 1PZN, chain A).

Residues E219_S208_ and D220_A209_ of *Pf*RadA are located between the FxxA and LFDE sites, with the BRC peptide interacting with equivalent *Hs*RAD51 residues. These were mutated to their human equivalents to create HumRadA2. There was a marked decrease in the thermal stability with this mutant, with the midpoint of thermal denaturation being lowered to 85 °C (Supplementary Fig. 2). This is possibly due to the disruption of an ionic interaction between E219_208_ and R270_254_, a hypothesis corroborated by the crystallographic analysis of HumRadA2 (Fig. 2a). Reduced stability, an unintended conssequence of mutagenesis, proved to be beneficial, as it allowed the measurement of full thermal denaturation curves during fragment screening by differential scanning fluorimetry (DSF) [37].

The structure of HumRadA2 also highlighted the need to introduce further mutations at the back of the Phe pocket (particularly V232Y_216_) in order to force I169M_158_ and K221M_210_ into the same conformation as observed in the *Hs*RAD51:BRC4 complex. Proper humanisation of the Phe pocket also required the removal of an insertion that lies just before V232_216_ (residues L226–P231).

Structural analysis confirmed that these mutations had granted a better resemblance to the *Hs*RAD51 FxxA pocket (Supplementary Fig. 1), making the resulting proteins HumRadA3 and HumRadA4 suitable for fragment-based approach to inhibitor design. Nevertheless, given the poor conservation of the LFDE pocket, those mutants still lacked the ability to bind BRC4. Interaction with BRC4 was essential both for the validation of the surrogates and for the development of a robust competition assay that would serve a screening campaign later. Therefore, we proceeded with a stepwise humanisation of the LFDE site and validated the resulting mutants using biophysical methods (as described in the next section).

**Fig. 3:**
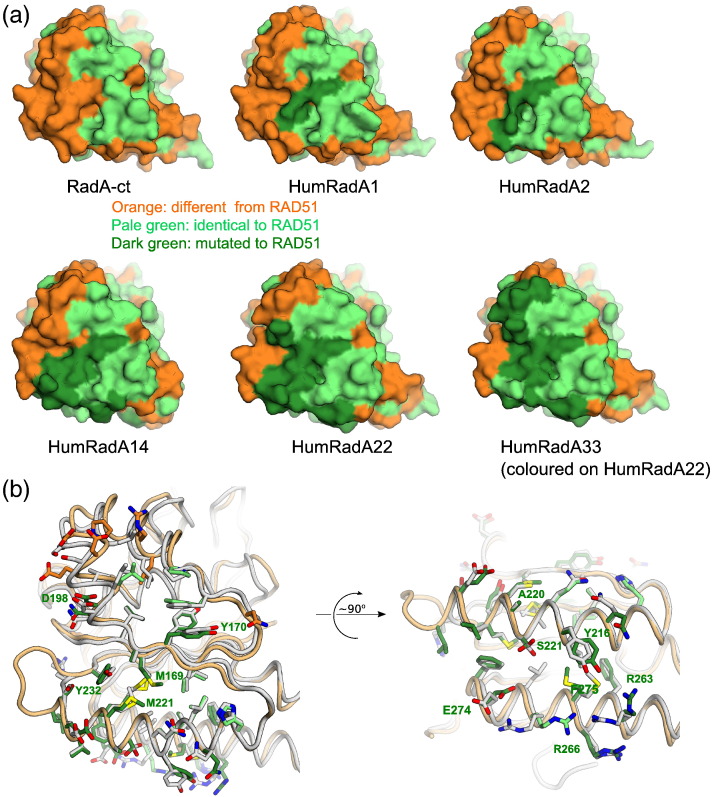
Characterisation and validation of humanised RadA mutants. (a) Surface representation of selected humanised RadA mutants with surfaces coloured as in Fig. 1B, with humanising mutations coloured in dark green. HumRadA33 as the final mutant is shown for completeness, but in the absence of its crystal structure, the humanisation is shown on the structure of HumRadA22. (b) Superposition of the HumRadA22 and human RAD51 in the FxxA (left) and LFDE (right) sites with each RadA residue coloured in green if it is identical to human residue. All RAD51 residues are shown in light grey, with the key residues discussed in the text labelled following RadA residue numbering.

Extensive humanisation made the protein progressively more difficult to purify and manipulate, but it often led to no significant change in the interaction with BRC4 until the right combination of mutations synergistically improved the affinity for BRC4. Many of the intermediate mutants were therefore not characterised in detail. The synergistic effect was particularly evident in mutant HumRadA14, with seven additional mutations compared to HumRadA5. HumRadA14 represents a “turning point” of the humanisation process as it is the first mutant to show detectable binding to BRC4 peptides, due to a combination of fine-tuning at the LFDE site and further re-shaping of the Phe pocket (mutations V168A_157_ and W170Y_179_; Supplementary Fig. 1). Charge mutations A226R_250_ and K198D_187_ (in HumRadA16 and 18, respectively) increased the BRC4 binding affinity and were combined in the following mutants. The humanisation series could have ended with HumRadA22, which displays a fully humanised BRC4 binding surface. Nevertheless, we extended the humanisation process further into the oligomerisation groove and “behind” the Ala pocket, in anticipation of the inhibitor design process expanding beyond the core of the FxxA binding site. This created our most humanised surrogate RadA, HumRadA33. Isothermal titration calorimetry (ITC) data confirmed that this last set of mutations ensured selectivity of HumRad33 for the human oligomerisation epitope over the archeal one (Table 1). The complete progress of converting RadA into RAD51-like protein is shown in Fig. 3a, with the illustration of the surface of HumRadA33 displaying continuous identity with RAD51. The crystal structure of HumRadA22 confirmed the success of the humanisation process and shows good agreement with the human RAD51 structure (Fig. 3b). Overall, the structures of the humanised RadA proteins are very similar to each other (Supplementary Fig. 2), and significant differences can only be seen in loops that are involved in either crystal packing, or are mobile and poorly defined.

The structures of those mutants, where the LFDE site has been humanised (HumRadA14 and 22), show good correlation with the human BRC4–RAD51 complex in this area for the backbone but some variation in the side-chain conformations (Fig. 3b and Supplementary Fig. 2e and f). Despite extensive efforts, we were unable to obtain a crystal structure of a complex of a HumRadA mutant with a BRC repeat, and differences in the BRC4 binding site are likely to reflect the unliganded state of our proteins.

Table 1 summarises all the mutants described in this paper. In the most humanised form of RadA, HumRadA33, a total of 32 residues have been mutated in and around the BRC4 binding site in RadA to resemble human RAD51. The resulting protein has 65% sequence identity to human RAD51 in the ATPase domain and 100% identity in the residues that interact with BRC4 and with the oligomerisation epitope (Fig. 3a and b).

Table 1 also shows how the humanisation progressively reduced protein stability. Nevertheless, even the most humanised mutants were still very thermostable, much more so than human RAD51 (Supplementary Fig. 2).

### Validation of humanised RadA mutants

The main objective of *Pf*RadA humanisation was to obtain a protein tailored for a fragment screening campaign that would then enable the development of inhibitors against the *Hs*RAD51:BRCA2 interaction. Therefore, the result of the humanisation had to be a protein that mimicked the human enzyme as closely as possible whilst providing a robust surrogate protein that was suitable for the biophysical and structural characterisations of the inhibitors.

Different peptidic tools were used to validate the progress of humanisation. A 16-aa peptide (RadA-OP) corresponding to the oligomerisation interaction epitope, as seen in the RadA heptameric crystal structure [26], was used as a probe for RadA-like interactions at the oligomerisation groove. A corresponding human oligomerisation peptide (RAD51-OP) was used to follow the progress of humanisation. The BRC4 peptide was used to assess the success of the humanisation of the full BRC interaction site. As a reference value, *Hs*RAD51 has been reported to have nanomolar affinity for BRC4 [40]; no previously published data were available for the interactions with the oligomerisation peptides.

The humanisation process was expected to progressively increase the affinity of the surrogate proteins for the RAD51-OP and, at the same time, weaken the binding for the RadA-OP. Such change in selectivity should be reflected into a different ability of these peptides to stabilise the surrogate proteins upon binding. DSF was used as a simple, high-throughput method to verify the initial hypothesis across the humanisation series. We have previously shown that different RadA and RAD51 constructs show significant stabilisation on binding to various nucleotides [38], and ATP was used as a positive control in DSF and to show that proteins are correctly folded (Fig. 2b). DSF is also one of the methods used in primary fragment screening, and this study also demonstrated the feasibility of this method to detect subtle affinity differences between ligands binding in this site. The results of the DSF screening were then validated using ITC by measuring the affinity of both RadA-OP and RAD51-OP for key mutants (Table 1 and Supplementary Fig. 3).

The binding of HumRadA proteins to RadA-OP (as monitored by DSF; Fig. 2b) was largely unaffected by mutations around the Phe pocket, and the affinity towards RadA-OP was reduced only when we fine-tuned the residues around the Ala pocket. In particular, DSF showed that the introduction of the K198_D187_ mutation reduced the Δ*T*_m_ by over 1 °C. In line with the expectations, the ITC experiments demonstrated that the affinity for the RadA-OP, being relatively robust for HumRadA1 (*K*_D_ = 1.8 ± 0.5 μM), was weakened by an order of magnitude with the most advanced versions of the surrogate system (HumRadA33, *K*_D_ = 14 ± 2 μM). Conversely, the affinity for the RAD51-OP increased compared to the early stages of the process (HumRadA1, *K*_D_ = 24 ± 5 μM), reaching submicromolar values for HumRadA33 (*K*_D_ = 0.8 ± 0.1 μM; Fig. 2d). We have also determined the crystal structure of HumRadA1 in complex with RadA-OP, which shows that the isolated oligomerisation peptide binds to the ATPase domain in a very similar way to what is observed in the heptameric ring structure of *Pf*RadA (Fig. 2f). The FxxA epitope straddles the central β-sheet, and the C-terminal end of the peptide folds into a short α-helix interacting with the oligomerisation groove. All the peptide residues that interact with the domain are in identical positions to those found in the oligomeric RadA.

Finally, it was necessary to demonstrate that the humanisation made *Pf*RadA mutants increasingly more competent for BRC4 binding as the peptide binding surface becomes more humanised. DSF, ITC, and fluorescence polarisation (FP) assay with a fluorescently labelled BRC4 peptide were used to probe the interaction with the key representative mutants (Fig. 2b–e). As predicted, the affinity towards BRC4 increased gradually, reflecting the progression of the humanisation (Fig. 2b, Table 1, and Supplementary Fig. 4). In agreement with the DSF results, the mutations introduced at the edge of the Ala pocket in HumRadA18 (K198D_187_/H199N_188_/I200V_189_) considerably facilitated the interaction with BRC4, increasing the affinity by an order of magnitude. A further gain in affinity was obtained in HumRadA20 by the introduction of A266_R250_ mutation in the LFDE site. The majority of these mutations aimed to reconstitute electrostatic interactions occurring between *Hs*RAD51 and the BRC4. For instance, the acidic group in D198_187_ probably reinstated a polar interaction with BRC residue S1528, whilst the A266R_250_ mutation reintroduced a basic residue that, in *Hs*RAD51, forms a salt bridge with the BRCA2 residue E1548 (Fig. 2c). The final humanised mutant, HumRadA33, has a *K*_d_ of 33 nM by ITC for BRC4 peptide (Fig. 2e).

The FxxA motif with the two hydrophobic residues is regarded the key epitope driving the formation of the BRC4:RAD51 complex. However, the results described in this section also highlight the functional relevance of the BRC4 C-terminal region, which establishes interactions ensuring the tight binding of the repeat to the recombinase [31]. Analysis of the roles of D187 and R250 in human RAD51 reveals how significant the contribution of these polar interactions is to BRC4 binding, both in the FxxA and the LFDE sites. These aspects of RAD51–BRC4 interaction have not been always appreciated and illustrate the importance of taking into account the whole interaction surface, and not just the hot-spots, when designing surrogate systems for PPIs.

### RadA/RAD51/RadA chimera

**Fig. 4:**
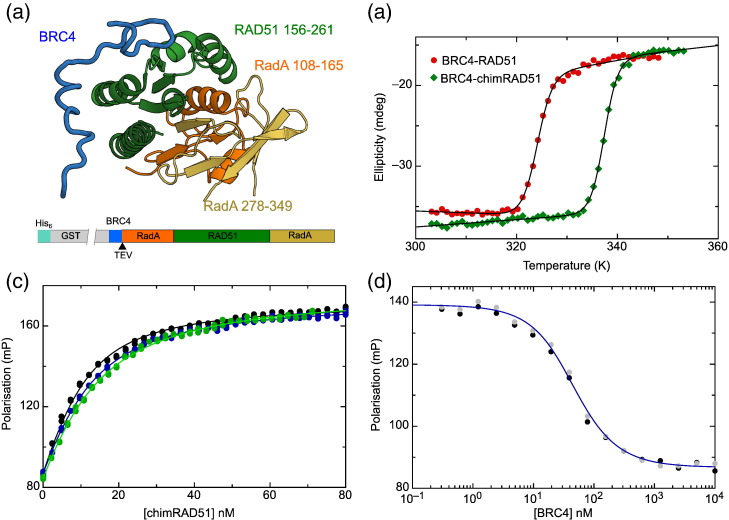
Design and validation of the chimeric RAD51 protein. (a) Domain structure of RAD51:BRC4 complex highlighting the parts that make up the chimeric protein. RadA N- and C-terminal parts are coloured orange and beige, respectively, with the central part of RAD51 in green. The diagram below shows the complete expression construct with His6-GST fusion, BRC4 protein with TEV cleavage site, and the ChimRAD51. (b) Thermal denaturation curves for BRC4–RAD51 (green) and BRC4–ChimRAD51 (red) following CD signal at 208 nm. (c) FP binding assay between ChimRAD51 and the fluorescently labelled BRC4 peptide. Each of the three curves and associated measurements represents an independent experiment. (d) Competition FP measurement using unlabelled BRC4 peptide to complete the Alexa Fluor 488-labelled peptide. Data are shown for two independent titrations, and the blue line is fitted to the average.

In parallel with the stepwise humanisation of RadA, we explored a second approach to creating a surrogate RAD51. In *Hs*RAD51, all the residues that form the BRC4 binding surface are contiguous in sequence within the ATPase domain, and the N- and C-terminal regions of this domain come together to form the rest of the globular structure (Fig. 4a). Also, the residues interfacing between the BRC4 binding segment and the N- and C-terminal parts of the ATPase domain are nearly identical between the RadA and RAD51. In the hope that RadA-derived sequences could stabilise the otherwise unstable human RAD51, an artificial archeal human chimeric construct (ChimRAD51) was designed, in which the entire BRC4 interacting part of human RAD51 (residues 156–261) is fused to the N- and C-terminal segments of the *Pf*RadA ATPase domain (residues 108–165 and 278–349, respectively; Fig. 4a). We envisaged that this engineered protein, in which only a small and distal part of the protein was derived from RadA, would retain human-like binding affinity towards BRC4 whilst benefiting from the stabilisation, due to the *Pf*RadA segments, and that this chimeric system could provide an independent comparison and validation of the humanisation approach.

Perhaps not surprisingly, when expressed in *Escherichia coli,* ChimRAD51 was insoluble, reflecting the difficulties in working with the human enzyme. As the original RAD51:BRC4 structure was determined using a fusion construct between the BRC4 repeat and C-terminal ATPase domain of RAD51, joined by a flexible linker [25], an analogous construct was made using ChimRAD51, with the introduction of a tobacco etch mosaic virus (TEV) protease cleavage site to facilitate the separation of ChimRAD51 from the BRC4 repeat (Fig. 4a). This construct, like the original BRC4–RAD51 fusion, was expressed solubly in *E. coli*, and the protein could be purified to homogeneity using either His-glutathione *S*-transferase (GST) or His-tag fusions.

To evaluate the success of our attempt to engineer a more stable protein, the BRC4–ChimRAD51 fusion was compared with the analogous BRC4–RAD51 fusion construct. Measured by CD, BRC4–ChimRAD51 fusion protein has an apparent *T*_m_ of 64 °C, a very significant 13 °C stabilisation over the human BRC4–RAD51 fusion (Fig. 4a). This demonstrated that the N- and C-terminal parts of the archeal protein increase the thermal stability of the protein significantly. However, it was still necessary to separate the BRC4 peptide and ChimRAD51. Unfortunately, the two remained stably associated after TEV cleavage and could not be separated without strong denaturing agents (Supplementary Fig. 5). In a final modification to the system, we weakened the BRC4:ChimRAD51 interaction by mutating the conserved phenylalanine 1524 in BRC4 to alanine. This mutant was still solubly expressed in bacteria, and we were able to separate the unliganded ChimRAD51 from the proteolytically released BRC4 peptide under native conditions in size-exclusion chromatography.

Using FP experiments, we could show that the BRC4 peptide binds to ChimRAD51 with nanomolar affinity (*K*_D_ = 4 ± 1 nM, Fig. 4c), a result that was confirmed by competition experiments (Fig. 4d). Both the direct binding isotherms and the competition assays were highly reproducible and invariant over time (≥ 1-h incubation; Supplementary Fig. 5) and were satisfactorily described by a single-site interaction model. Following final validation *via* ITC (Supplementary Fig. 5b), it was concluded that the ChimRAD51 retained the ability to tightly bind BRC4 in a simple 1:1 fashion, thus providing a faithful mimicry of the recombinase–peptide binding event in the absence of any oligomerisation process. The 10-fold higher affinity for ChimRAD51 in comparison to full-length *Hs*RAD51 [40] can be explained by the monomeric state of ChimRAD51 and the lack of the N-terminal domain, which has been proposed to participate in BRC4 binding. We are therefore confident that ChimRAD51 is a *bona fide* surrogate of *Hs*RAD51 in its unliganded form, and it became our primary screening tool. Being more stable than its human counterpart and practicable for FP and ITC experiments, this protein is suitable for fragment-based approaches, where various biophysical techniques are used to screen fragment libraries and analyse newly created molecules in iterative cycles of validation, design, and optimisation. In the context of a drug discovery programme, we have used ChimRAD51 to establish an FP-based competition assay in 384-well format for a rapid and reliable screening of molecules capable of displacing BRC4 from RAD51 (Supplementary Fig. 5c).

### Oligomeric surrogate proteins

Full-length RAD51 and RadA form oligomers in solution, and in the case of RAD51, these oligomers can be disrupted by BRC4 [32]. A faithful surrogate of human RAD51 should show the same behaviour, if provided with the structural determinants that induce oligomerisation. As a final validation, we generated full-length variants of HumRadA22 (flHumRadA22) and ChimRAD51 (flChimRAD51), by adding to them the missing N-terminal parts (with the oligomerisation sequences) from RadA and RAD51, respectively. Dynamic light scattering (DLS) experiments (Supplementary Fig. 6) confirmed that the full-length surrogates formed oligomers in solution. When BRC4 repeat peptide was added into the samples, the oligomeric proteins showed dose-dependent dissociation and shift to smaller molecular size. This demonstrated that both approaches in creating human-like surrogates were also able to reproduce the functional effect of the BRC4 repeat on an oligomeric protein.

### Development of a crystallographic platform using humanised RadA

**Fig. 5:**
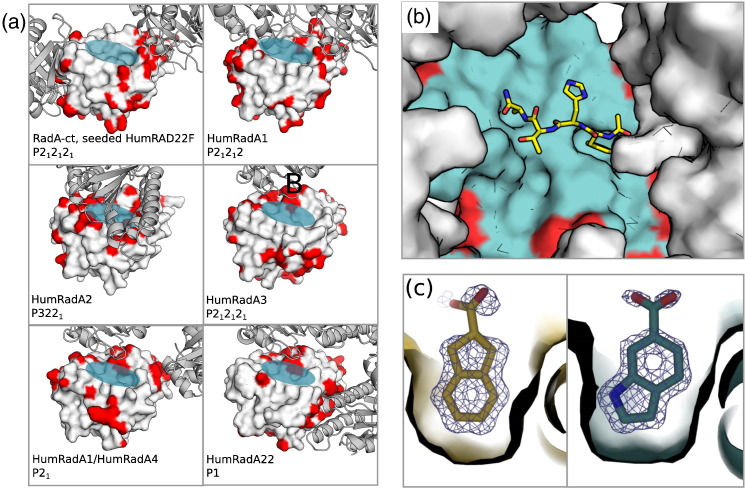
Crystallographic system using humanised RadA. (a) Crystal packing in various crystal forms obtained for humanised RadA proteins. Protein with molecular surface represents the solved structure, and the ribbon diagrams are the symmetry-related molecules in the crystal lattice next to the FxxA site. Contacts between the proteins in the lattice are coloured red on the surface of the central molecule. The transparent blue ellipse indicates the FxxA binding site in the central molecule. (b) FxxA binding site with superimposed FHTA peptide in the wild-type-like crystal form of the HumRadA22F mutant. (c) Two examples of fragment hits from crystallographic fragment cocktail screen using HumRadA22F crystals, binding in the Phe pocket (thin surface outline). Final 2F_obs_-F_calc_c electron densities are rendered at 1σ.

We were unable to crystallise ChimRAD51, and many of the more advanced humanised HumRadA constructs failed to yield crystals of sufficient quality for structure-guided drug design. We therefore returned to the earlier surrogates (HumRadA1–3; Fig. 5) that crystallised readily in conditions similar to those originally identified for monomeric RadA-ct. As we were targeting the PPI between RAD51 and the BRC4 peptide, the mutated residues were invariably on the surface of the protein. As a result, mutants often crystallised in different crystal forms, and accessibility of the FxxA binding pocket varied significantly (Fig. 5a). For instance, mutant HumRadA1 crystallises in one monoclinic (P2_1_) and two orthorhombic (P2_1_2_1_2_1_ and P2_1_2_1_2) space groups with little similarity in crystal contacts, but we could control the obtained crystal forms through cross-seeding with seeds from crystals of different mutants.

To test the suitability of a given crystal form for soaking experiments, we used the tetrapeptide FHTA (corresponding to the FxxA motif of the BRC4 repeat) as a sizeable test compound of 516 Da. It was possible to soak the peptide at relatively low concentrations (2–5 mM) into RadA-ct crystals and into the orthorhombic form of HumRadA1, as verified by structures determined using these crystals [30]. The ability to soak peptides into the crystals gave confidence that soaking for fragment-based drug design would be successful.

Whilst it was possible to obtain apo crystals of the fully humanised mutants (HumRadA22), the crystals were difficult to grow and handle and hence not well suited for drug discovery purposes. As the reduced crystallisability seemed to be a consequence of humanisation of the LFDE site, we decided to reverse these mutations and created two surrogates (HumRadA22F and HumRadA33F) for crystallographic purposes. These proteins were fully humanised in the extended FxxA binding area and in the oligomerisation groove, but these proteins contained the RadA-like LFDE site (see also Fig. 1 for reference to the relative position of these structural elements). They were easier to purify than their corresponding fully humanised mutants HumRadA22 and HumRadA33, and they readily formed crystals that yielded high-quality diffraction data and tolerated up to 10% dimethyl sulphoxide (DMSO) during small-molecule soaking experiments.

Of all the RadA variants examined, it turned out that the original wild-type RadA-ct construct crystallised in the most suitable crystal form, with a fully open FxxA binding site (Fig. 5a). These crystals were used as seeds to drive HumRadA22F protein into the same crystal form. This cross-seeding, which we had used with other and more closely-related mutants before, was successful and provided a system where the entire target site was fully humanised, open, and unobscured by crystal contacts (Fig. 5a). These crystals of HumRadA22F were finally validated by soaking the FHTA tetrapeptide into the crystal, which demonstrated that drug-sized molecules can diffuse into these crystals and bind to the target site (Fig. 5b).

### Crystallographic fragment screen

To demonstrate the robustness of our crystallographic system for drug discovery purposes, we used HumRadA22F crystals to perform a direct X-ray crystallographic screening of a library containing 352 fragments, and we soaked 88 cocktails of 4 fragments into these crystals. We were able to collect diffraction data from all of the soaked crystals to typically 1.6–1.8 Å resolution or better (up to 1.3 Å), despite handling crystals in a high-throughput fashion. Approximately 50% of structures showed the binding of an unspecific ring system in the Phe pocket, of which we classified 15 as hits, that is, 17% of the crystal structures showed a discernible compound bound, which equates to an overall hit rate of 4%. For 3 of these 15 structures, it was not possible to uniquely identify directly from the density which of the cocktailed chemical entities had bound. These would need deconvolution of the mixture by each of the fragments as singletons. The remaining 12 hits could be clearly identified from the density, which is owed to structurally diverse cocktailing. Two representative examples of well-defined fragments in the Phe pocket are shown in Fig. 5c. Notably, we did not observe fragment binding to any additional sites, particularly to the Ala pocket, which is presumably too shallow and devoid of hydrogen-bonding opportunities to provide enough binding energy for small molecule to bind on its own. The soaking procedure did not seem to have a significant impact on crystal quality, suggesting that no high-affinity fragment binding sites are obscured by crystal contacts.

## Discussion

At the start of our drug discovery project to design inhibitors against human recombinase RAD51, we encountered several problems. The natural oligomerisation of RAD51 occludes the target site, and the only known monomeric construct of RAD51 was a fusion protein comprising RAD51 and a peptide that binds to that same site with high affinity. It was therefore necessary to create a non-oligomeric form of RAD51 with an open binding site. Whilst it was easy to design a deletion mutant that would not oligomerise, the resulting protein was not stable and it was not possible to purify an active form for screening and validation.

In order to overcome these difficulties, we decided to take advantage of the high thermal stability of an archeal ortholog, RadA from *P. furiosus,* which is structurally highly related to human RAD51 [26]. Using two parallel approaches, we have created monomeric proteins that show a significant increase in thermal stability and reduced propensity for aggregation, such that they can be produced in large quantities for structural and biophysical analysis. A fully humanised BRC repeat binding site has been introduced into these mutants, either through residue-by-residue mutagenesis or through replacing the large parts of the archeal protein with the equivalent region of the human protein. The resulting humanised proteins bind the BRC4 repeat with low nanomolar affinity in a 1:1 fashion and are suitable for ligand binding studies using a number of biophysical approaches. The humanised RadA proteins, being very stable at high concentrations over a long time scale, were employed in several biophysical assays and in crystallography, enabling the determination of hundreds of structures of inhibitors in complex with a target surrogate. In addition, a reproducible FP screening assay was developed using a chimeric ChimRAD51 protein. Together, the chimera and humanised RadA provided all the protein–target forms to support a structure-driven, fragment-based drug discovery programme that generated high-potency RAD51 inhibitors (manuscripts in preparation).

Surrogate systems are not unknown in drug discovery, but they are generally represented by either orthologs or paralogs with high sequence identity to the target, thus requiring little mutagenesis to become a reliable replacement of the original protein. Kinases, frequently the targets of inhibitor development campaigns, offer good examples of chimeric surrogates. Chimeras of PKA-PKB [41], PKA-S6K1 [42], and PDK1-PKCζ [43] have been used to study the activity and inhibition of PKB, S6K1, and PKCζ, respectively, but in all cases, only a few mutations in the active site were needed for the creation of these chimeras. In the case of the RAD51 PPI, creating an unliganded system with an open binding side suitable for drug discovery presents additional challenges due to the high affinity of the partner peptide and the apparent obligate binding required to keep RAD51 stable. The methodology described here should enable work on other PPI systems, which have otherwise been intractable to date.

Chimeric proteins have been used before to promote the crystallisation of difficult targets and to study their interactions with relevant inhibitors. Domain swapping between the bacterial and mouse proteins made it possible to determine the structure of the cytoplasmic domain of the eukaryotic Kir3.1 membrane channel, whereas the original mammalian protein failed to yield crystals [44]. Loop swapping led to the crystallisation of a chimeric phosphodiesterase catalytic domain in complex with sidenafil and with an inhibitory peptide, revealing important aspects in the mechanism of phosphodiesterase 6 inhibition [45]. The same strategy was used to improve the affinity of the Melanoma IAP protein for Caspase-9, and the resulting chimera was then successfully used for the development of Melanoma IAP antagonists [46], [47]. Finally, humanisation of the active site of rat fatty acid hydrolase generated a surrogate that was easy to produce and displayed the same inhibitor sensitivity profile as its human counterpart [48].

Whilst archeal proteins are often used as models for human enzymes, to our knowledge, this is the first example where an archeal protein has been used as a basis for developing a surrogate system for drug discovery. In contrast to the examples quoted above, the starting point for our humanisation/chimerisation approach was an archeal ortholog that displayed a relatively low sequence identity with the target: 53% overall, and even less in the target site. Nevertheless, this thermostable protein provided a “scaffold” that could be successfully transformed into a monomeric protein and then reprogrammed for BRC4 binding, without compromising higher stability with respect to its human counterpart. Those characteristics enabled us to overcome the limitations presented by RAD51 and thus justified the choice of such a distantly related protein.

Whilst domain swapping with thermostable orthologs is not unprecedented, this strategy does not always lead to clear stabilisation of the mesophilic counterpart [49]. Success of this approach was reported with RNase H, where a stabilisation of approximately 10 °C was achieved by replacing a portion of the *E. coli* enzyme with its thermophilic counterpart [50].

Although archeal orthologs are not necessarily available for all potential drug targets, we believe this is an attractive approach when such homologues exist and particularly worth pursuing when the original target proves difficult to handle. Also, this approach is by no means limited to archeal surrogates. A similar approach can also be applied to bacterial proteins and orthologs from thermostable mycobacteria like *Mycobacterium hassiacum* and *Mycobacterium thermoresistibilis* , whose proteins have been proposed [51], [52] and used [53], [54] as surrogates for proteins from *Mycobacterium tuberculosi*s. As more genomic data become available from diverse species, the sequence space from which we can mine novel sequences with more suitable properties for biochemical and structural analysis is continuously increasing. We are not aware of algorithms that are designed for selecting ortholog surrogates for biochemical analysis, but this could be a valuable resource for the research community. Currently, the search for possible surrogate is done by collecting orthologous sequences and analysing them for conservation in relevant parts of the sequence. Tools for analysing the stability of proteins are available [55] and could be used as part of the search criteria, but eventually, the choice of a surrogate will be based on several criteria, and the final outcome will be a result of an empirical trial-and-error process.

**Fig. 6:**
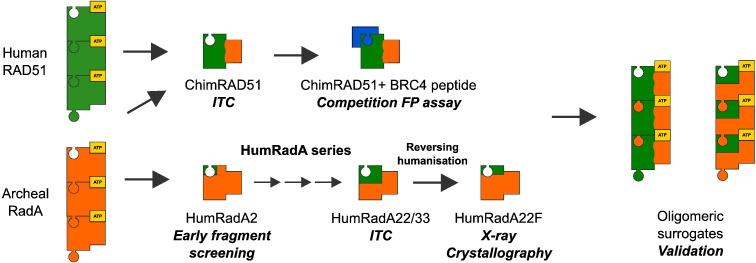
Summary of RAD51 surrogate systems. The “jigsaw puzzle pieces” with green (RAD51) and orange (RadA) colouring illustrate the progress of humanisation and ChimRAD51 development. Different mutants have served different purposes in our drug discovery programme, as labelled in the diagram. This summary illustrates our experience that more than one protein or protein form is needed to enable all the diverse analyses required for modern structure-guided drug discovery.

Whilst we took a stepwise approach to the humanisation of the BRCA2 binding site on RadA in order to build an understanding of the relationship between protein sequence and properties such as stability and ligand binding, the generation of a suitable protein tool could be easily completed in fewer steps if it focused solely on generating a useful protein mutant. At the same time, our work presented here shows that one surrogate protein might not be suitable for all the diverse assays and analyses that are needed in a fragment- and structure-guided drug discovery project. Understanding of the changes introduced by stepwise humanisation of RadA has facilitated the development of efficient surrogates for all the biophysical and structural methods employed in the project, and this alone validates the approach we have taken (Fig. 6). In addition to providing a surrogate system to support our structure-guided drug design, we have also gained invaluable insight into the biology of the target. We also report the first affinity measurements of BRC4 binding to the monomeric form of RAD51, and our stepwise humanisation has allowed us to elucidate the effects of individual residues in the interaction between RAD51 and BRC4 and its own oligomerisation peptides. Given the difficulties in working with human RAD51, our surrogate approach has proven its value and potential not just in approaching inhibitor studies but also in providing valuable insight into the target, highlighting the critical role of polar residues in RAD51–BRC4 interaction.

## Materials and Methods

### Cloning

All humanised RadA expression constructs were cloned by PCR using Phusion polymerase starting from the wild-type *Pf*RadA sequence as the template (ENA: AAC34998) and cloned as *Nco*I-*Xho*I fragments into the T7-based *E. coli* expression vector pBAT4 [56]. The monomeric RadA protein described here starts at amino acid 108 of the native *Pf*RadA protein with an initiation methionine added to its N terminus. Site-directed mutagenesis, including the deletion of loop L2, was performed using the overlapping primer extension method, and the resulting mutant constructs were cloned in a similar way into the vector pBAT4. The ATPase domain of *Hs*RAD51 (encoding residues 97–339, preceded by sequence encoding for MetGly) was cloned into the vector pBAT4 using *Nco*I and *Hind*III restriction sites. Untagged ChimRAD51 was constructed by inserting the DNA encoding for residues 156–261 of *Hs*RAD51 (Uniprot: Q06609) between the DNA sequence encoding for residues 108–165 and 278–349 of *Pf*RadA (Uniprot: O74036) and by cloning this chimeric DNA construct into the vector pBAT4 using *Nco*I and *Xho*I restriction sites. For the fusion constructs that include a BRC repeat, the DNA sequence coding for the BRC4 repeat of BRCA2 (amino acid residues 1517–1551; Uniprot: P51587) was fused with a short linker coding for a canonical TEV protease recognition site, and the resulting sequence was cloned into the vector pGAT2 using *Bam*HI and *Nco*I restriction sites. The ChimRAD51 was then inserted in this as a *Nco*I-*Xho*I fragment to create a fusion construct coding for the protein construct His–GST–BRC4–TEV–ChimRAD51. For the generation of full-length ChimRAD51 and HumRadA22 constructs, sequences coding for residues 1–97 of human RAD51 and 1–107 of *Pf*RadA were fused to the 5′ end of the respective monomeric constructs, and the resulting sequences were cloned into the vector pBAT4. All constructs were verified by automated sequencing. Full-length RAD51 surrogates were created by adding the human N-terminal domain and oligomerisation sequence (residues 1–96) to ChimRAD51 and the corresponding parts of *Pf*RadA (residues 1–107) to HumRadA22, creating flChimRAD51 and flHumRadA22, respectively. These were cloned into pBAT4 *E. coli* expression vector.

All sequences were verified by sequencing, and the amino acid sequences for all the proteins described in this work are provided at the end of the Supplementary Data in FASTA format.

### Protein expression and purification

All proteins were expressed in *E. coli* strain BL21(DE3) carrying pUBS520 plasmid [57]. Cells were grown to an OD_600_ of 0.8–1.0. For humanised RadA constructs, expression was induced by the addition of 400 μM IPTG and was allowed to proceed for 3 h at 37 °C. For ChimRAD51 constructs, the growth temperature was reduced to 15 °C for 1 h before the induction of expression by the addition of 400 μM IPTG and overnight incubation. Cells were pelleted by centrifugation, resuspended in ultrapure water, and stored at − 20 °C.

Cells expressing HumRadA proteins were lysed in 20 mM MES and 0.5 mM EDTA (pH 6.0) using an Emulsiflex C5 homogeniser. The lysate was heated to 65 °C for 15 min to denature *E. coli* proteins, with the exception of the mutants HumRadA14–33, which were not heat treated as they were more prone to aggregation. Lysates were centrifuged at 15,000*g* for 30 min, and the clarified supernatant was loaded onto a 5-ml HiTrap SP HP column (GE Healthcare). Bound protein was eluted using a linear gradient to 500 mM NaCl in 10 column volumes. RadA mutants typically eluted at 250–300 mM NaCl. Peak fractions were pooled, concentrated, and further purified by gel filtration using a Superdex 75 16/60 pg or 26/60 pg column (GE Healthcare) equilibrated with 20 mM Mes, 100 mM NaCl, and 0.5 mM EDTA (pH 6.0), with the exception of mutants HumRadA5–33, which were purified in 20 mM Ches and 200 mM NaCl (pH 9.5). Peak fractions were pooled, concentrated to 0.5 mM using 10-kDa MWCO PES centrifugal concentrators (Millipore), flash frozen in liquid N_2_, and stored in aliquots at − 80 °C. The typical yield was 15–20 mg of protein from a 1 L culture of *E. coli*.

ChimRAD51, expressed as a fusion with His-GST-BRC4, was purified initially using Ni Sepharose 6 Fast Flow (GE Healthcare). This was followed by gel filtration on a Superdex 75 16/60 pg column (GE Healthcare) in the arginine, glutamate, and phenylalanine (“REF”) buffer [50 mM Hepes/Na (pH 8.0), 200 mM KCl, 100 mM arginine, 100 mM glutamate, and 100 mM phenylalanine] [58]. This buffer was designed to improve the long-term stability of the protein before and after TEV protease cleavage, and it allowed the protein to be flash frozen with liquid N_2_ and stored at − 80 °C until later use. ChimRAD51 was then cleaved with TEV protease to release the monomeric protein and was separated from His-GST-BRC4 fragment by repeating the gel-filtration step. For optimal separation of the two proteins, the loading concentration was adjusted to obtain a GST-BRC4 peak of 1.5–2.0 AU (on a 1 cm path length cell) at elution. For applications that demanded the highest purity (e.g., ITC and crystal trials), purified ChimRAD51 monomer was loaded by syringe onto a 1 mL Ni-Sepharose FF column equilibrated with REF buffer plus 20 mM imidazole (pH 8.0), and the flow through was collected. This optional step removed the remaining traces of His-tagged TEV protease and His-GST-BRC4. Before performing ITC or FP experiments, the ChimRAD51 (1–2 mL, 30–60 μM) was dialysed overnight at 4 °C against 20 mM K/phosphate (pH 8.0) and 192 mM KCl, changing the buffer three times. The protein could be stored in this buffer at 4 °C for up to a week with ~ 10% loss of material due to aggregation per day; experimental artefacts arising from the presence of aggregates were ameliorated by regular filtration of the material, followed by redetermination of the concentration with correction for the apparent absorbance from light scattering.

Full-length surrogates flChimRAD51 and flHumRadA22 were expressed in soluble form in *E. coli* together with His6–MBP–BRC4 fusion constructs and were purified by a combination of metal ion and heparin affinity chromatographies (Moschetti *et al*., in preparation). Both proteins eluted early from Superdex 200 10/300 column, as reported for the oligomeric RAD51 (data not shown).

### Peptide synthesis

Peptides were synthesised by PNAC Service (Department of Biochemistry) using standard FMOC chemistry, except for unlabelled BRC4 repeat, which was expressed in *E. coli.* All peptides were acetylated in the N-terminus and amidated in the C-terminus. The sequences were as follows: RadA oligomerisation peptide Ac-NLGTFMRADEYLKKRA-NH_2_, RAD51 oligomerisation peptide Ac-VPMGFTTATEFHQRRS-NH_2_, and BRC4 repeat Ac-CKEPTLLGFHTASGKKVKIAKESLDKVKNLFDEKEQ-NH_2_. Fluorescently labelled BRC4 repeat was prepared by reacting the synthetic peptide with an excess of maleimide-Alexa Fluor 488 dye, followed by reversed-phase chromatography using 4.6 × 250 mm Ace C18 300 Å column. The *E. coli*-produced BRC4 repeat that was expressed as a His_6_–GB1 fusion with HRV 3C protease cleavage site before the peptide, purified by Ni^2 +^ affinity chromatography, digested with HRV 3C protease overnight and free peptide, and separated by reversed-phase chromatography using a 2 ml Source RPC column (GE Healthcare). Purified peptide was analysed and quantified the same way as the synthetic peptides. The resulting peptide had the sequence GPGSMSKEPTLLGFHTASGKKVKIAKESLDKVKNLFDEKEQGSS. All peptides were characterised by mass spectrometry to confirm the correct molecular masses and incorporation of labels (when appropriate), and their concentrations were determined by quantitative amino acid analysis (PNAC service, Department of Biochemistry).

### ITC

ITC was performed using a Microcal itc200 instrument at 25 °C. Based on the different isoelectric points of the mutants, proteins were dialysed either into 50 mM Tris–HCl (pH 7.5), 150 mM NaCl, and 5 mM MgCl_2_ (RadA-ct, HumRadA2, HumRadA3, HumRadA5, HumRadA16, and HumRadA18) or into 50 mM Tris–HCl (pH 8.5), 250 mM NaCl, and 5 mM MgCl_2_ (HumRadA14, HumRadA20, HumRadA22, and HumRadA33). Experiments typically involved titrating 20 μM of protein in the sample cell with 200 μM of BRC4 peptide in the syringe (30 μM of protein and 300 μM of ATP for ITC involving ATP binding). The raw ITC data were then fitted using a single-site binding model using the Microcal ITC LLC data analysis program in the Origin 7.0 package.

### FP assay

FP binding and competition experiments were performed at 25 °C in 100 mM Tris (pH 7.5), 200 mM KCl, and 1.25% (vol/vol) glycerol. The samples were loaded onto Costar 96-well half-area black microplates, and fluorescence data were recorded using a PheraStar plate reader (BMG) equipped with polarisation filters (excitation at 485 nm, emission at 520 nm). The concentration of Alexa Fluor 488-labelled BRC4 peptide was constant at 10 nM, and the concentration of each protein titrant was adjusted based on the dissociation constant observed in trial experiments. Binding isotherms were fitted to a standard quadratic equation for single-site binding.

Competitive binding experiments were set up with a constant assay concentration of 10 nM Alexa Fluor 488-labelled BRC4 peptide, and a constant assay concentration of protein determined to give approximately 80–90% saturation of binding in direct binding experiments. A serial dilution of each competitor peptide was prepared and mixed with the protein and labelled peptide, and the resulting competitive binding isotherms were measured and fitted using the expression described by Wang [59].

### Thermal stability analysis using DSF

DSF was conducted using a BioRad CX96 or CFX real-time PCR instruments. *Pf*RadA mutants were buffer exchanged into 20 mM Hepes-KOH, with same conditions of pH and ionic strength as that for ITC (see above). Each sample had a final volume of 100 μl and was prepared with 25 μl of 1:500 diluted fluorescent SYPRO Orange dye, 65 μl of protein solution at 4 μM final concentration, and either 10 μl of 20 mM Hepes-KOH (for the unliganded control sample) or 10 μl of a ligand. The latter was prepared at the following stock concentrations: 2.6 mM for each peptide (RadA-OP, RAD51-OP, or BRC4), or 10 mM MgCl_2_ and 10 mM ATP. Temperature was increased from 37 °C (310 K) to 95 °C (368 K) at a rate of 1 °C per minute. Raw data was resampled and converted to the negative of the first derivative (− dRFU/dT) before the *T*_m_ was determined as the minimum of the negative derivative.

### CD spectroscopy

We prepared 300 μl of 10 μM *Pf*RadA mutants in 20 mM Hepes at either pH 7.5 (HumRadA2, HumRadA5, HuRadA16, and HumRadA18) or at pH 8.5 (HumRadA22) with a total ionic strength adjusted to 150 mM with NaCl. CD spectra were measured in a Hellma Quartz Precision cell with a 1-mm light path using Jasco J-815 CD Spectrometer. The ellipicity (mDeg) of each mutant was measured at 222 nm wavelength from 40 °C (313 K) to 95 °C (368 K), with a temperature ramp rate of 1 °C per minute. All curves and *T*_m_ values were fitted and derived using the Pro Fit software using a CD thermal denaturation equation.

### DLS

Average particle size and polydispersity index were determined by DLS at 25 °C with a Zetasizer Nano Z system (Malvern Instruments, U.K.) and were analysed using DTS software package for Windows (version 7.11, Malvern, U.K). Each protein was diluted to 15 μM concentration in a final volume of 70 μL of 100 mM Tris (pH 7.5) and 200 mM KCl. Particle scattering was recorded for the apo protein and then for the same sample after the addition of BRC4 repeat (see Peptide synthesis) in 1:1, 1:5, and 1:10 M excesses with respect to the protein concentration.

### Crystallography

Monomeric RadA was screened for suitable crystallisation conditions using Qiagen Classic and PEG suite screens and Emerald Biosystems Wizard Screens. The loop2 deletion mutants crystallised in most cases with polyethylene glycol (PEG) as a precipitant, and the final refined conditions used for structure determination are listed in Table S1.

Crystals were cryocooled in liquid N_2_, and diffraction data were collected at ESRF (Grenoble, France), Soleil (Saint-Aubin, France) and Diamond Light Source (Harwell, UK) synchrotron radiation sources. The data were processed with XDS [60] or autoPROC [61] and where applicable; scaling was performed using Scala or Aimless [62], [63]. Initial phases were obtained by molecular replacement, initially using the structure of oligomeric RadA (PDB**:** **1PZN**) as a search model using CCP4 program Amore [64]. Later on, the high-resolution structures of the monomeric proteins were used as search models for new mutant structures. Structures were refined with Refmac5 [65], phenix.refine [66], and autoBUSTER [67]. Manual real-space refinement was performed using Coot [68]. For details on crystallographic data collection and refinement statistics, please see Table S1.

### Crystallographic fragment screen

The fragment library was obtained from Zenobia Fragments (San Diego, USA). Unliganded crystals of HumRadA22F were grown by mixing 2 μl of a reservoir solution (16–20% PEG8000, 80 mM sodium cacodylate, 190 mM calcium acetate, and 20% glycerol) with 2 μl of protein solution (as described above) and 0.5 μl of seed stock obtained from previous crystals. These were soaked in cocktails of four compounds, where each compound was present at a final concentration of 5 mM in 10% DMSO. The crystals were soaked for approximately 24–48 h in a hanging-drop vapour-diffusion setup. For this, 4.5 μl of the reservoir containing 80 mM sodium cacodylate, 160 mM calcium acetate, 18% PEG8000, and 20% glycerol was mixed with 0.5 μl of fragment cocktail in 100% DMSO. To avoid the loss of the thin and often not well visible HumRadA22F crystals in conditions with compound precipitation, we added the fragment solution from the side of the drop, whilst the crystal was manually transferred to the opposite side of the drop, and the mixing of the drop was avoided as far as possible. Structure determination and initial ligand fitting were automated using Pipedream [67], using the apo structure as a search model. Subsequent refinement was performed with autoBUSTER and phenix.refine. Ligand restraints were calculated using grade [69] in combination with a mySQL/ChemAxon Instant JChem database (version 16.2.15.0, 2016, ChemAxon†) containing the chemical fragment structures.

### Accession numbers

Coordinates and structure factors have been deposited in the Protein Data Bank with accession numbers: 5FOS, 5LB2, 5LBI, 5L8V, 5LB4, 5KDD, 5J4L, 5JEE, 5JED, 5JEC, 5JFG, 5J4H, 5J4K.

## Acknowledgements

We would like to thank the large team of scientists from the Departments of Biochemistry and Chemistry and from the MRC Cancer Unit who have participated in the RAD51 drug discovery project. We are grateful for access to X-ray data collection facilities at various synchrotrons: Diamond Light Source (beamlines I02, I03, I04, I04-1 and I24, under proposals mx315, mx6889, mx7141, mx9007), ESRF (beamline ID23-1), and Soleil (beamline Proxima1) and for the support provided by the beamline staff. We would like to thank Drs. Dimitri Chirgadze and Katherine Stott of the Crystallographic and Biophysics facilities at the Department of Biochemistry for providing access and support at these facilities. The Protein and Nucleic Acid Facility at the Department of Biochemistry is thanked for mass spectrometric and amino acid analyses. This work was funded by Wellcome Trust Translational (080083/Z/06/Z) and Seeding Drug Discovery Initiative (91050/Z/10/Z) awards.

## Figures and Tables

**Table 1:** Details of HumRadA mutants and summary of binding data

Mutant	Mutations (all constructs carry also deletion of the L2 loop)	*T*_m_ (°C)	BRC4 affinity (nM)/FP	RadA-OP affinity (μM)/ITC	RAD51-OP affinity (μM)/ITC
HumRadA1	I169M,Y201A, V202Y, K221M	94.3	n.d.	1.8 ± 0.5	24 ± 5
HumRadA2	I169M, Y201A, V202Y, **E219S**, **D220A**, K221M	82.1	weak	3.4 ± 0.3	n.d.
HumRadA3	I169M, Y201A, V202Y, E219S, D220A, K221M, **I222M, V223M, V232Y**	n.d.	n.d.	n.d.	n.d.
HumRadA4	I169M, Y201A, V202Y, E219S, D220A, K221M, I222M, deletion of 227–231,V232Y, K233A	n.d.	n.d.	n.d.	n.d.
HumRadA5	I169M, Y201A, V202Y, **L213Q, V215L, Q216Y**, E219S, D220A, K221M, **D267M, L274E, Y275F**	80.8	weak	n.d.	n.d.
HumRadA14	**V168A**, I169M, **W170Y,** Y201A, V202Y, L213Q, V215L, Q216Y, E219S, D220A, K221M, **I222M**, **K223V, L225S, V232Y, H264F,** D267M, L274E, Y275F	78.4	670 ± 12	n.d.	n.d.
HumRadA16	V168A, I169M, W170Y, Y201A, V202Y, L213Q, V215L, Q216Y, E219S, D220A, K221M, I222M, K223V, L225S, V232Y, K263R, H264F, **A266R**, D267M, L274E, Y275F	77.2	294 ± 6	n.d.	n.d.
HumRadA18	V168A, I169M, W170Y, **K198D, H199N, I200V**, Y201A, V202Y, L213Q, V215L, Q216Y, E219S, D220A, K221M, I222M, K223V, L225S, V232Y, K263R, H264F, D267M, L274E, Y275F	76.1	10.7 ± 0.35	n.d.	n.d.
HumRadA20	V168A, I169M, W170Y, K198D, H199N, I200V, Y201A, V202Y, L213Q, V215L, Q216Y, E219S, D220A, K221M, I222M, K223V, L225S, V232Y, K263R, H264F, A266R, D267M, L274E, Y275F	75.5	3.90 ± 0.15	n.d.	n.d.
HumRadA22	V168A, I169M, W170Y, **I182L**, K198D, H199N, I200V, Y201A, V202Y, L213Q, V215L, Q216Y, E219S, D220A, K221M, I222M, K223V, L225S, V232Y, K263R, H264F, A266R, D267M, L274E, Y275F	74.0	6.20 ± 0.30	n.d.	n.d.
HumRadA33	**S167K**, V168A, I169M, W170Y, **N175G**, I182L, **R183L**, **D192S, P193G D194S, E195D**, K198D, H199N, I200V, Y201A, V202Y, L213Q, V215L, Q216Y, E219S, D220A, K221M, I222M, K223V, L225S, V232Y, K263R, H264F, A266R, D267M, L274E, Y275F	n.d.	n.d.	14 ± 2	0.8 ± 1
HumRadA22F	V168A, I169M, W170Y, I182L, K198D, H199N, I200V, Y201A, V202Y, K221M	n.d.	n.d.	n.d.	n.d.
HumRadA26F	S167K, V168A, I169M, W170Y, N175G, I182L, R183L, K198D, H199N, I200V, Y201A, V202Y, E219S, K221M, I222M,	n.d.	n.d.	n.d.	n.d.
HumRadA28F	S167K, V168A, I169M, W170Y, N175G, I182L, K198D, H199N, I200V, Y201A, V202Y, L213Q, V215L, Q216Y, E219S, K221M, I222M, K223V, L225S, V232Y, K263R, H264F, A266R, D267M, L274E, Y275F	n.d.	n.d.	n.d.	n.d.
HumRadA33F	S167K, V168A, I169M, W170Y, N175G, I182L, R183L, D192S, P193G D194S, E195D, K198D, H199N, I200V, Y201A, V202Y, E219S, K221M, I222M, K223V, V232Y	n.d.	n.d.	n.d.	n.d.

A list of all the humanised RadA mutants described in the article, with the mutations they carry (numbering as per *Pf*RadA sequence) and the details of thermal stability (measured by DSF) and affinities towards BRC4 and RadA and RAD51 oligomerisation (OP) peptides, measured by ITC. It is worth noting the decreasing thermal stability as humanisation progresses and the increasing affinity towards BRC4 and RAD51-OP peptides. Mutations introduced for the first time are highlighted in bold. n.d. – not determined.

## Appendix A Supplementary Data

Supplementary figures and tables. Image 2

## Footnotes

† http://www.chemaxon.com
