# Supplementary material for: Engineering Archeal Surrogate Systems for the Development of Protein–Protein Interaction Inhibitors against Human RAD51
Source: J Mol Biol. 2016 Nov 20;428(23):4589–607. doi: 10.1016/j.jmb.2016.10.009 (PMC5117717; doi:10.1016/j.jmb.2016.10.009)
Supplement: Supplementary file 1 — Supplementary figures and tables. [file mmc1.pdf]

Supplementary material

Moschetti *et al.* Engineering archeal surrogate systems for the development of protein-protein interaction inhibitors against human RAD51

Contents

Supplementary material 1

Supplementary Table 1. 2

Supplementary Figure 1 4

Supplementary Figure 2. 5

Supplementary Figure 3. 6

Supplementary Figure 4. 7

Supplementary Figure 5. 8

Supplementary Figure 6. 9

Supplementary Figure 7. 10

Sequences of all the proteins........ 11

Supplementary Table 1.

Crystallographic data collection and refinement statistics

	HumRadA1 +RadA-OP	HumRadA2	HumRadA3	HumRadA4	HumRadA14	HumRadA22	HumRadA22F
PDB accession number	5FOS	5LB2	5LBI	5L8V	5LB4	5KDD	5J4L
**Data collection:**							
Beamline	DLS I03	DLS I02	DLS I04	DLS I03	Soleil Proxima-1	DLS I02	DLS I04-1
X-ray wavelength [Å]	0.9700	0.9796	0.9799	0.9700	0.97903	0.9795	0.9173
**Data processing:**							
Spacegroup	$P\ 2_1\ 2_1\ 2_1$	$P\ 3_2\ 2\ 1$	$P\ 2_1$	$P\ 2_1\ 2_1\ 2_1$	$P\ 2_1$	P 1	$P\ 2_1\ 2_1\ 2_1$
Unit cell (a, b, c) [Å]	40.79, 61.78, 87.22	90.04, 90.04, 67.86	37.35, 77.98, 39.81	37.61, 71.61, 74.84	39.51, 37.81, 69.45	37.85, 42.58, 86.44	40.48, 61.06, 87.91
(α,β,γ) [°]	90.0, 90.0, 90.0	90.0, 90.0, 120.0	90.0, 117.5, 90.0	90.0, 90.0, 90.0	90.0, 92.4, 90.0	78.6, 85.5, 63.6	90.0, 90.0, 90.0
Resolution limits [Å]	50.0-1.35	50.0-2.10	40.0-1.43	50.0-1.50	45.0-1.98	84.7-1.99	30.0-1.13
(High resolution shell) [Å]	(1.43-1.35)*	(2.23-2.10)*	(1.52-1.43)*	(1.59-1.50)*	(2.10-1.98)*	(1.994-1.988)*	(1.20-1.13)*
Number of molecules in ASU	1	1	1	1	1	2	1
No of total/unique reflections	232321 / 47394	203161 / 18731	126928 / 36018	232974 / 32967	50568 / 14305	55356 / 31086	454090 / 148188
Multiplicity	4.9 (4.8)	10.9 (10.9)	3.5 (3.0)	7.0 (7.0)	3.5 (3.3)	1.8(1.8)	3.1 (1.6)
R_{merge}	0.107 (0.586)	0.056 (0.394)	0.052 (0.257)	0.093 (0.544)	0.141 (0.700)	0.130 (0.286)	n/a
R_{meas}	0.043 (0.153)	0.051 (0.644)	0.045 (0.226)	0.063 (0.668)	0.088 (0.635)	0.183 (0.405)	0.051 (0.485)
I/σI	16.3 (2.7)	29.46 (4.20)	20.51 (5.56)	20.40 (3.25)	10.67 (2.29)	5.0 (2.1)	14.8 (2.03)
CC ½	n/a	n/a	n/a	n/a	n/a	n/a	99.9 (75.2)
Completeness [%]	96.3 (98.6)	99.7 (99.4)	97.1 (91.4)	99.8 (99.2)	99.1 (98.0)	95.2 (94.5)	93.7 (67.5)
**Refinement:**							
R_{work}/R_{free}	0.141 / 0.182	0.222/ 0.252	0.164 / 0.193	0.164 / 0.193	0.229 / 0.260	0.230 / 0.250	0.123/ 0.145
No. of unique/free reflections	47391 / 2399	18731 / 964	36017 / 1798	32966 / 1670	14304 / 705	30909 / 1566	78955 / 3954
R.m.s deviations:							
bond lengths [Å]	0.009	0.010	0.010	0.010	0.010	0.011	0.009
bond angles [°]	1.30	1.11	1.11	1.16	1.10	1.20	1.13
Ramachandran analysis:							
Favoured / Allowed/ Outliers	232 / 2 / 0	214 / 3 / 0	212 / 2 / 0	215 / 3 / 0	198 / 6 / 1	422 / 4 / 0	222 / 0 / 0
Number of atoms:							
Protein / Solvent / Hetero	1949 / 290 / 53	1718 / 41 / 0	3443 / 270 / 271	3472** / 240 / 245	1702 /44 / 44	3428 /239 / 259	1933 / 298 / 1
Mean/ Wilson B-factor [$Å^2$]	18.4 / 18.5	50.1 / 44.4	18.0 / 14.9	20.3 / 17.2	38.4 / 29.7	25.1 / 20.8	15.2 / 9.2
Crystallisation condition:	8% PEG1000, 0.1 M Na/KPO_4, pH 6.2	25% PEG6000, 0.1M MES pH 7.0	4-8% PEG1000, 0.1M Na/KPO_4 pH 5.6	12% PEG20000, 0.1 M MES pH 6.5	25% PEG4000, 0.1 M Tris pH 8.5	20% PEG3350, 0.2M $MgSO_4$	20% PEG8000, 0.08 M Na Cacodylate pH 6.5, 0.16M Ca Acetate, 18% glycerol
Cryo-/soaking condition:	10 % glycerol	10% glycerol	10% glycerol				

Supplementary table 1 (continued)

	HumRad26F	HumRad28	HumRadA33F	HumRadA22F +FHTA peptide	HumRadA22F + fragment A5	HumRadA22F + fragment H7
PDB accession number	5JEE	5JED	5JEC	5JFG	5J4H	5J4K
**Data collection:**						
Beamline	DLS I24	DLS I24	ESRF ID23-1	DLS I04	DLS I04	DLS I04
X-ray wavelength [Å]	0.9686	0.9686	0.9763	0.9794	0.9795	0.9795
**Data processing:**						
Spacegroup	P 2_1 2_1 2_1	P 2_1	P 3_1 2	P 2_1 2_1 2_1	P 2_1 2_1 2_1	P 2_1 2_1 2_1
Unit cell (a, b, c) [Å]	50.58, 50.60, 74.27	37.77, 72.10, 41.93	90.75, 90.75, 102.51	40.52, 59.27, 88.14	40.46, 61.12, 87.77	40.44, 61.21, 87.74
(α,β,γ) [°]	90.0, 90.0, 90.0	90.0, 115.9, 90.0	90.0, 90.0, 120.0	90.0, 90.0, 90.0	90.0, 90.0, 90.0	90.0, 90.0, 90.0
Resolution limits [Å]	74.3-1.49	37.7-1.33	78.6-2.34	59.3-1.77	61.1-1.37	87.7-1.35
(High resolution shell) [Å]	(1.492-1.487) *	(1.337-1.332) *	(2.348-2.340) *	(1.775-1.769) *	(1.378-1.374) *	(1.350-1.346) *
Number of molecules in ASU	1	1	2	1	1	1
No of total/unique reflections	211473 / 31823	214016 / 45801	225489 / 21095	114496 / 21412	238051 / 44591	293070 / 46330
Multiplicity	6.6 (4.5)	4.7 (3.2)	10.7 (10.6)	5.3 (5.5)	5.3 (4.4)	6.3 (5.3)
R_{merge}	0.063 (0.844)	0.093 (1.086)	0.193 (1.462)	0.082 (0.841)	0.056 (0.662)	0.045 (0.707)
R_{meas}	0.068 (0.958)	0.103 (1.304)	0.202 (1.537)	0.091 (0.931)	0.062 (0.755)	0.050 (0.785)
I/σI	17.5 (2.2)	7.6 (2.1)	9.9 (1.5)	13.5 (2.2)	17.7 (2.0)	24.9 (2.1)
CC ½	n/a	0.992/0.575	0.997(0.652)	0.998 (0.744)	0.999 (0.650)	1.000 (0.733)
Completeness [%]	99.3 (87.6)	99.3 (99.1)	100.0 (100.0)	100.0 (100.0)	96.5 (76.7)	94.4 (58.5)
**Refinement:**						
R_{work}/R_{free}	0.194/ 0.218	0.135/ 0.171	0.199/ 0.239	0.174/ 0.192	0.132/ 0.170	0.129/ 0.164
No. of unique/free reflections	31579/ 1617	43497/ 2263	21043/ 1079	21337/ 1080	44537/ 2241	46275/ 2322
R.m.s deviations:						
bond lengths [Å]	0.010	0.012	0.010	0.011	0.004	0.008
bond angles [°]	1.26	1.58	1.20	1.10	0.87	1.12
Ramachandran analysis:						
Favoured / Allowed / Outliers	206 / 5 / 1	227 / 1 / 0	445 / 5 / 0	219 / 1 / 0	221 / 0 / 0	221 / 0 / 0
Number of atoms:						
Protein / Solvent / Hetero	1746 / 87 / 88	1923 / 201 / 22	3478 / 89 / 12	3638** / 141 / 209	3822** / 276 / 13	3711** / 241 / 30
Mean/Wilson B-factor [$Å^2$]	33.5 / 22.5	21.2 / 15.5	39.8 / 45.6	28.9 / 23.9	18.3 /12.0	19.9 / 13.1
Crystallisation condition:	15% PEG2000, 0.1M Na Acetate pH 4.6	40% MPD, 5% PEG8000, 0.1 M Na Cacodylate pH 6.5	22% PEG3350, 0.1 M BisTris pH5.0, 0.2 M $LiSO_4$	18% PEG8000, 0.08 M Na Cacodylate pH 6.5, 0.16 M Ca Acetate 20% glycerol	18% PEG8000, 0.08 M Na Cacodylate pH 6.5, 0.16M CaAcetate 20% glycerol	18% PEG8000, 0.08 M Na Cacodylate pH 6.5, 0.16 M Ca Acetate, 20% glycerol
Cryo-/soaking				5 mM FHTA, 10% DMSO	4 x 5 mM ligand (cocktail), 10% DMSO	4 x 5 mM ligand (cocktail), 10% DMSO

* values in parenthesis are for the highest resolution shell, as indicated

** these values include riding hydrogen atoms . n/a not available

Supplementary Figure 1

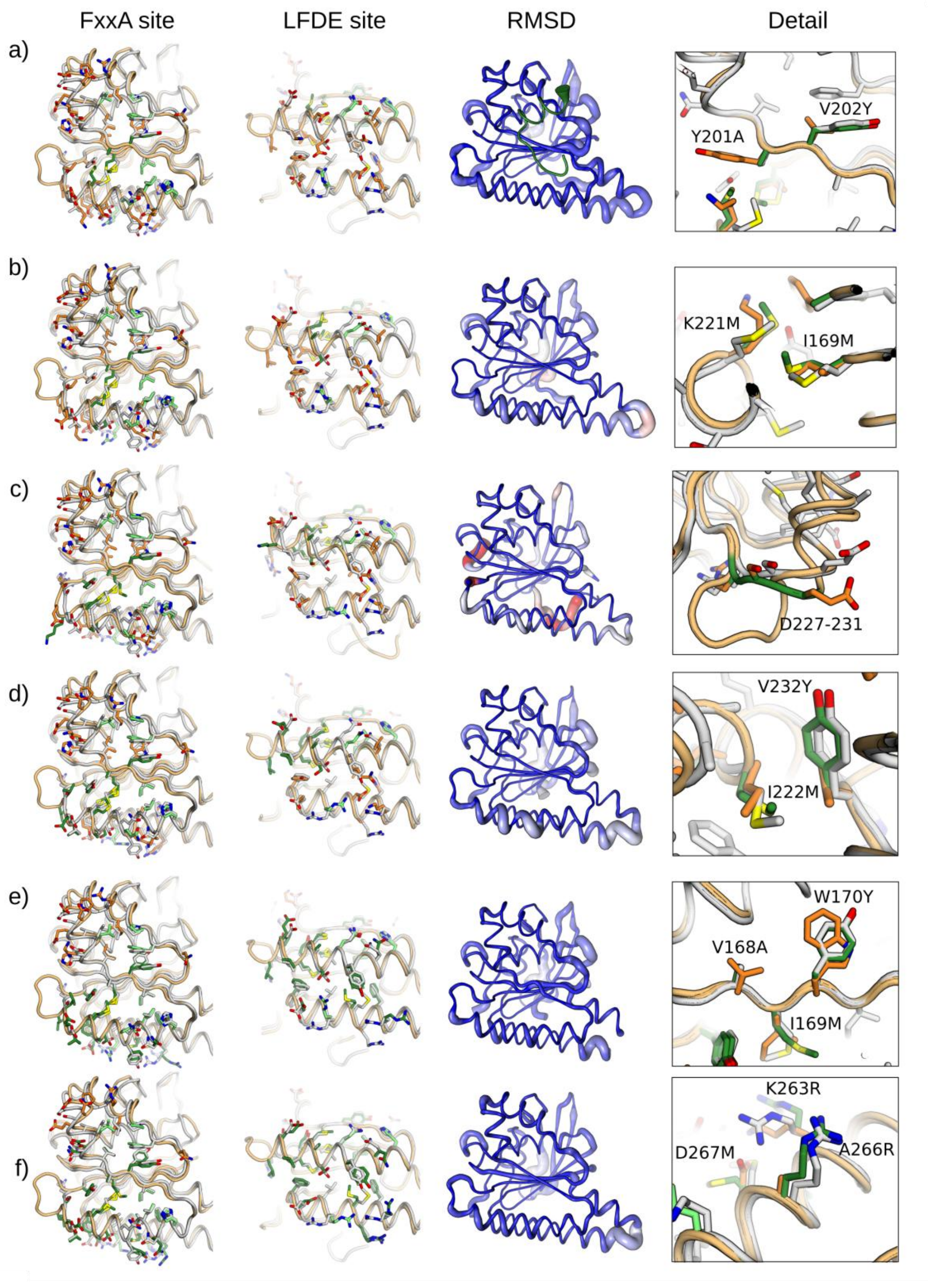

Structures of HumRadA mutants. In all the panels the two left-most figures show an overlay of the HumRadA mutant (brown backbone) and human RAD51 (white backbone and white carbon atoms). HumRadA residues are coloured with brown if different from RAD51, pale green if naturally identical and with dark green if humanised to RAD51-like. The third panel shows the superpositioning of the HumRadA mutant with human RAD51 with the RMSD between the structures shown as the thickness of the ribbon. The right-most panel shows a detail of a humanising mutation introduced in that particular mutant. (a) HumRadA1, (b) HumRadA2, (c) HumRadA3, (d) HumRadA4, (e) HumRadA14 and (f) HumRadA22

Supplementary Figure 2.

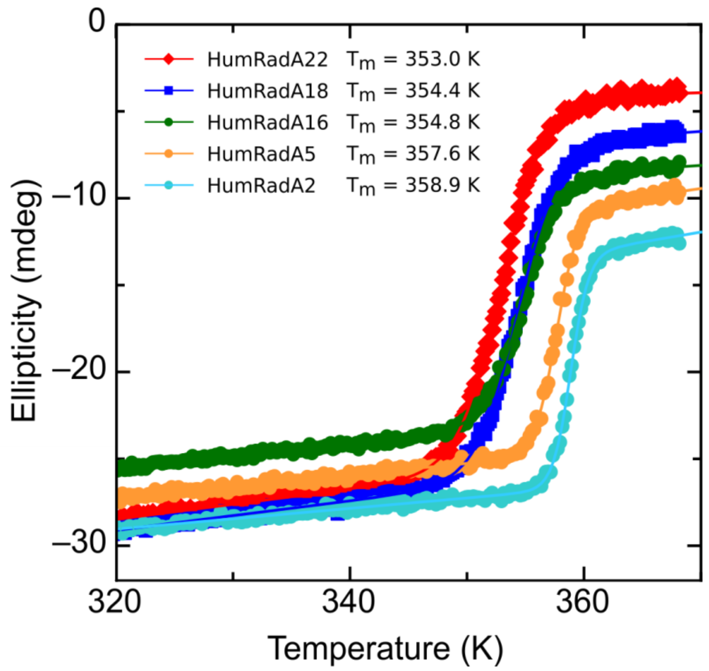

Thermal stability of HumRadA mutants. Analyses of the thermal stability of the HumRadA mutants, as labelled in the graph, using CD spectroscopy and following the change of molar ellipticity at 222 nm.

Supplementary Figure 3.

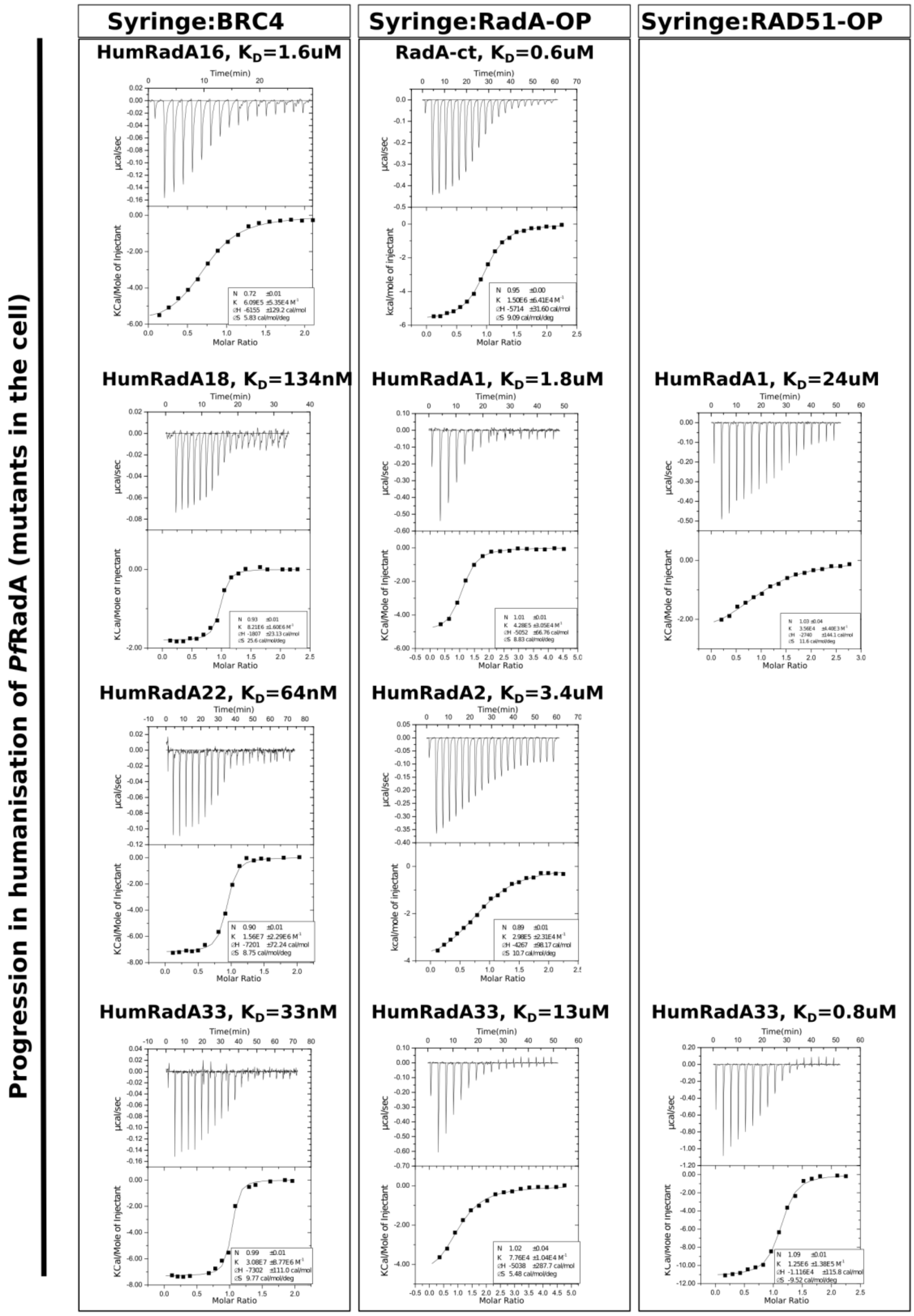

ITC measurements of peptide binding to HumRadA mutants. In each graph the top panel shows the baseline corrected raw data from the titration and the bottom graph shows the integrated heats of binding for each injection of the peptide (black squares) and the solid line the fit into single binding site mode model. The first column shows the data for titration of BRC4 peptide into different mutants, as labelled in the figure. The middle columns shows the binding to RadA oligomerisation peptide (RadA-OP) to different mutants and the right panel showing the binding data for RAD51 oligomerisation peptide (RAD51-OP).

Supplementary Figure 4.

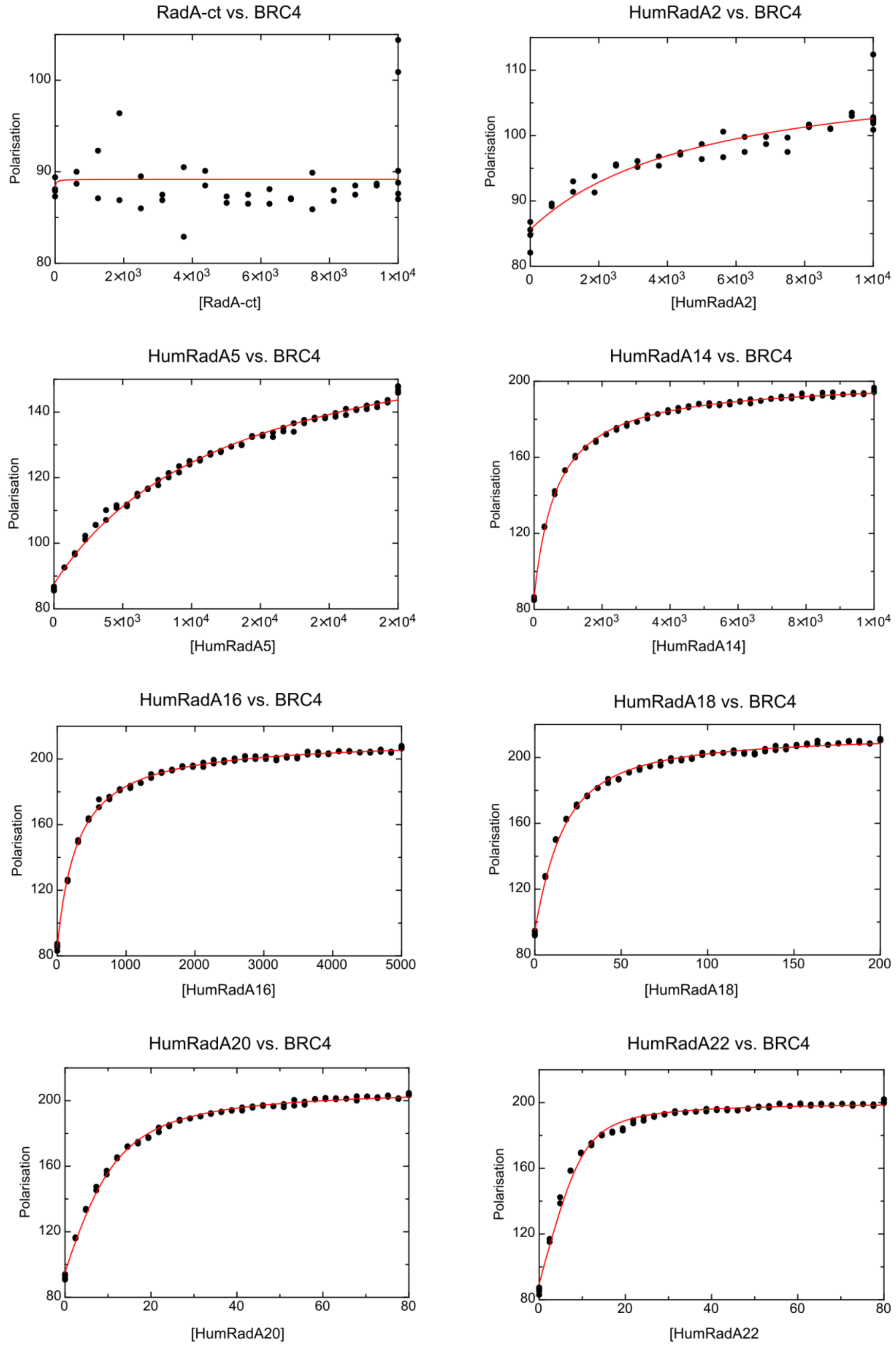

FP binding data for BRC4 peptide interaction with HumRadA mutants. Each graph represents titration of the indicated HumRadA mutant into Alexa Fluor 488-labelled BRC4 peptide, as described in Materials and Methods. All affinities are listed in Table 1 of the main article.

Supplementary Figure 5.

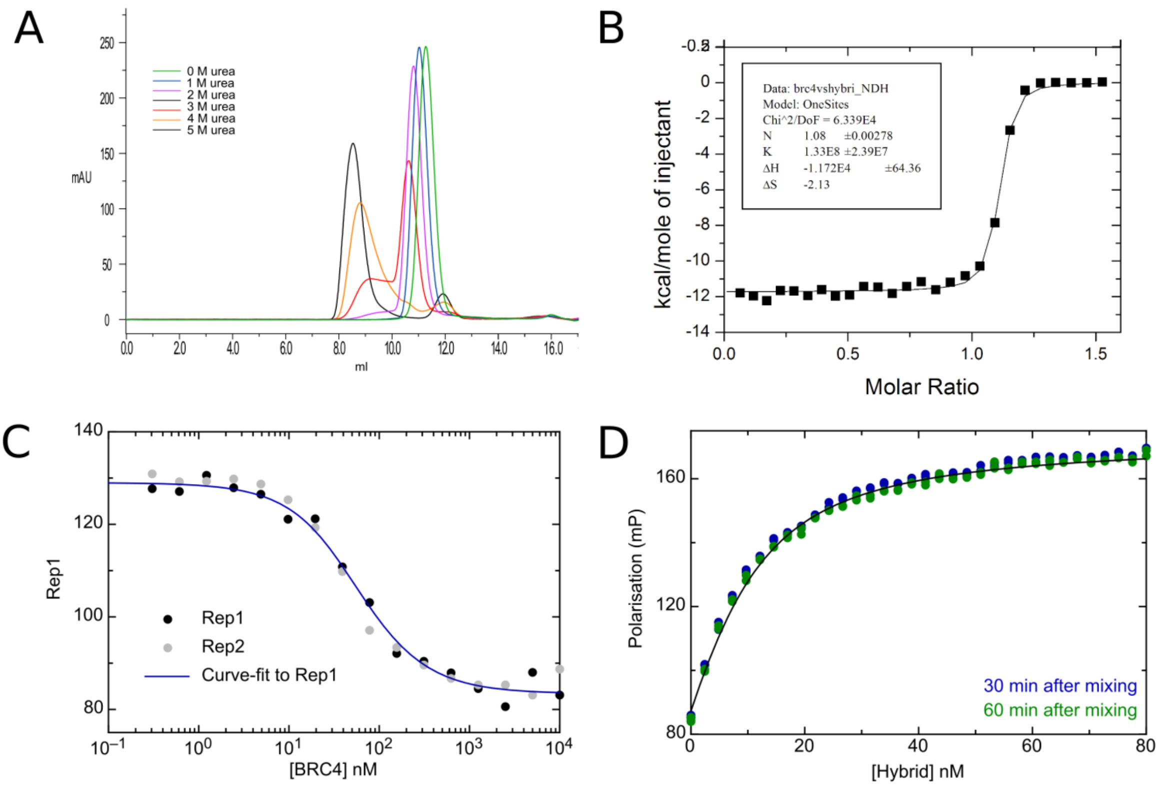

Analyses of ChimRAD51 and development of displacement FP assay. (A) Size exclusion chromatography of TEV-cleaved BRC4-RAD51 complex in increasing urea concentration in the running buffer, as indicated in the figure. Only at 5 M urea, when RAD51 denatures (as shown by elution close to void volume due to large hydrodynamic radius of the main peak), do the BRC4 peptide and RAD51 separate. (B) ITC titration of BRC4 peptide into ChimRAD51, showing a K_d of 6 nM (C) Competition FP assay in 384-well format using unlabelled BRC4 to compete Alexa Fluor 488-labelled BRC4 tracer, showing suitability of the assay to be miniaturised. (D) Stability ChimRAD51 in BRC4 binding assay is demonstrated by two FP titrations measured at 30 (blue) and 60 (green) minutes after peptide and protein were mixed.

Supplementary Figure 6.

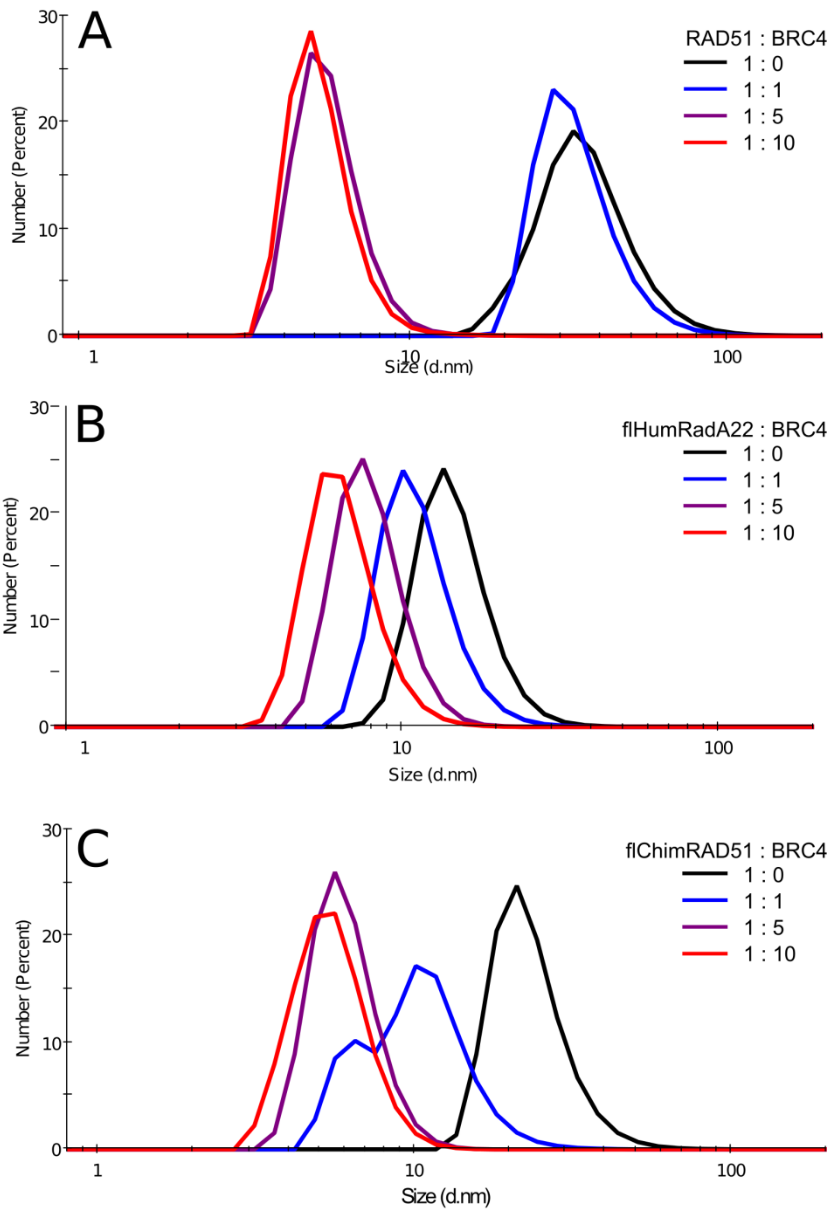

Dynamic light scattering analyses of full-length RAD51 and its surrogate forms. (A) Size distribution (expressed as a percentage of total particles in the sample) of purified human RAD51 protein alone (black line, average hydrodynamic radius: 32.7nm) or in the presence of increasing concentration of BRC4 peptide (blue, purple and red lines), as indicated in the graph as a stoichiometric ratio. (B) Similar data as in A, but using full-length HumRadA22 (average hydrodynamic radius 13.5 nm) (C) Like A, but using full-length ChimRAD51 (average hydrodynamic radius 21.0 nm).

Supplementary Figure 7.

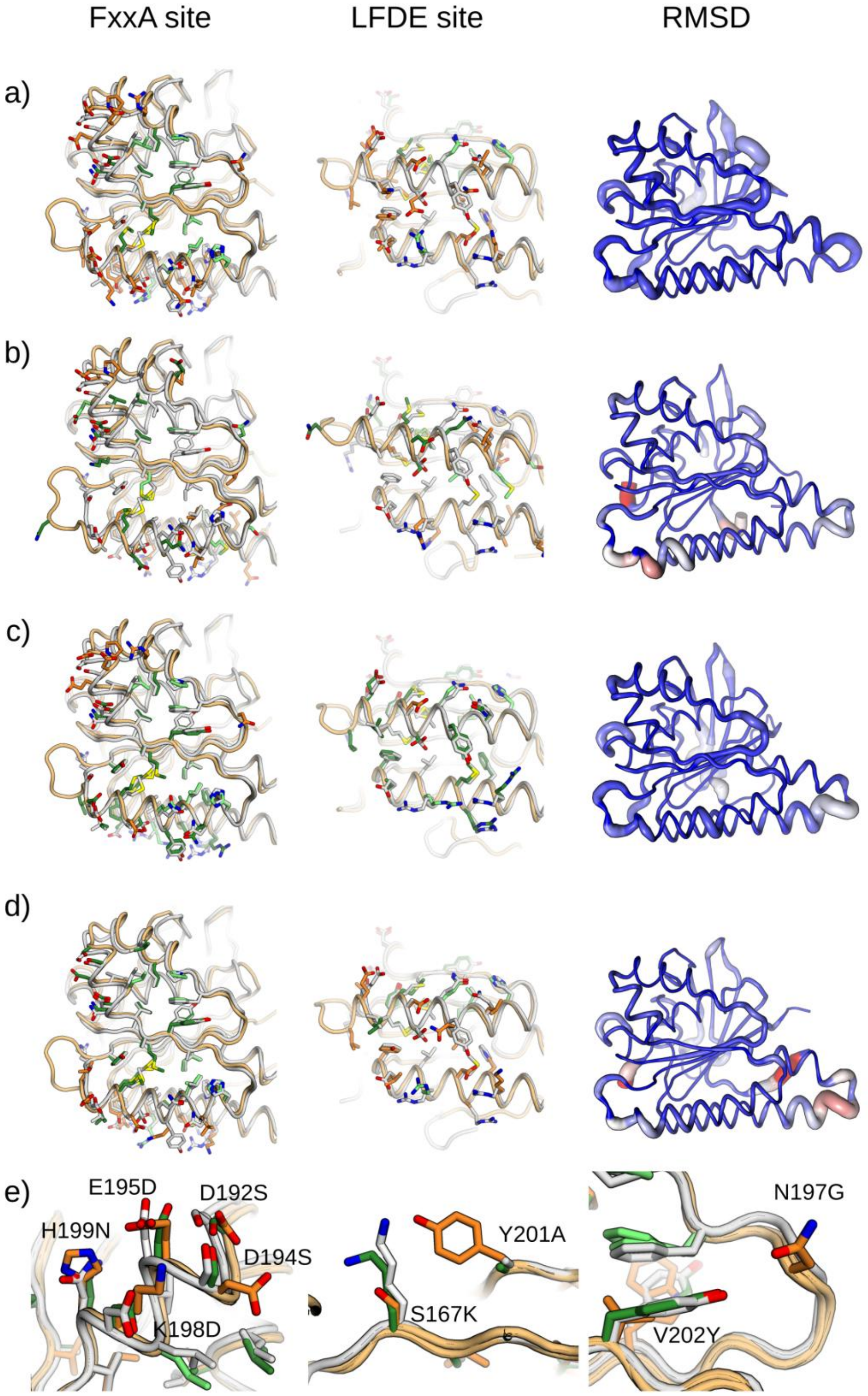

Comparison of the crystallographic surrogates with RadA and human RAD51. The two left-most figures in panels (a-d) show an overlay of the HumRadA mutant (brown backbone) and human RAD51 (white backbone and white carbon atoms). HumRadA residues are coloured with brown if different from RAD51, pale green if naturally identical and with dark green if humanised to RAD51-like. The third panel shows superpositioning of the HumRadA with human RAD51 with RMSD between the structures shown as thickness of the ribbon. (a) HumRadA22F, (b) HumRadA26F, (c) HumRadA28F, (d) HumRadA33F. (e) Details of extended humanisation mutations introduced into the FxxA site and oligomerisation groove of HumRadA33F (and HumRadA33), coloured as in (a-d)

Sequences of all the proteins

FASTA formatted amino acid sequences of all the proteins described in this paper, including wild type *P. furiosus* RadA and human RAD51.

>PfRadA-ct
MATIGRISTGSKSLDKLLGGGIETQAITEVFGEFGSGKTQLAHTLAVMVQLPPEEGGLNG
SVIWIDTENTFRPERIREIAQNRGLDPDEVLKHIYVARAFNSNHQMLLVQQAEDKIKELL
NTDRPVKLLIVDSLTSHFRSEYIGRGALAERQQKLAKHLADLHRLANLYDIAVFVTNQVQ
ANGGHILAHSATLRVYLRKGKGGKRIARLIDAPHLPEGEAVFSITEKGIED
>HumRadA1
MATIGRISTGSKSLDKLLGGGIETQAITEVFGEFGSGKTQLAHTLAVMVQLPPEEGGLNG
SVMWIDTENTFRPERIREIAQNRGLDPDEVLKHIAYARAFNSNHQMLLVQQAEDMIKELL
NTDRPVKLLIVDSLTSHFRSEYIGRGALAERQQKLAKHLADLHRLANLYDIAVFVTNQVQ
ANGGHILAHSATLRVYLRKGKGGKRIARLIDAPHLPEGEAVFSITEKGIED
>HumRadA2
MATIGRISTGSKSLDKLLGGGIETQAITEVFGEFGSGKTQLAHTLAVMVQLPPEEGGLNG
SVMWIDTENTFRPERIREIAQNRGLDPDEVLKHIAYARAFNSNHQMLLVQQASAMIKELL
NTDRPVKLLIVDSLTSHFRSEYIGRGALAERQQKLAKHLADLHRLANLYDIAVFVTNQVQ
ANGGHILAHSATLRVYLRKGKGGKRIARLIDAPHLPEGEAVFSITEKGIED
>HumRadA3
MATIGRISTGSKSLDKLLGGGIETQAITEVFGEFGSGKTQLAHTLAVMVQLPPEEGGLNG
SVMWIDTENTFRPERIREIAQNRGLDPDEVLKHIAYARAFNSNHQMLLVQQASAMMVELL
NTDRPYKLLIVDSLTSHFRSEYIGRGALAERQQKLAKHLADLHRLANLYDIAVFVTNQVQ
ANGGHILAHSATLRVYLRKGKGGKRIARLIDAPHLPEGEAVFSITEKGIED
>HumRadA4
MATIGRISTGSKSLDKLLGGGIETQAITEVFGEFGSGKTQLAHTLAVMVQLPPEEGGLNG
SVMWIDTENTFRPERIREIAQNRGLDPDEVLKHIAYARAFNSNHQMLLVQQASAMMKESR
YALLIVDSLTSHFRSEYIGRGALAERQQKLAKHLADLHRLANLYDIAVFVTNQVQANGGH
ILAHSATLRVYLRKGKGGKRIARLIDAPHLPEGEAVFSITEKGIED
>HumRadA5
MATIGRISTGSKSLDKLLGGGIETQAITEVFGEFGSGKTQLAHTLAVMVQLPPEEGGLNG
SVMWIDTENTFRPERIREIAQNRGLDPDEVLKHIAYARAFNSNHQMQLLYQASAMIKELL
NTDRPVKLLIVDSLTSHFRSEYIGRGALAERQQKLAKHLAMLHRLANEFDIAVFVTNQVQ
ANGGHILAHSATLRVYLRKGKGGKRIARLIDAPHLPEGEAVFSITEKGIED
>HumRadA14
MATIGRISTGSKSLDKLLGGGIETQAITEVFGEFGSGKTQLAHTLAVMVQLPPEEGGLNG
SAMYIDTENTFRPERIREIAQNRGLDPDEVLKHIAYARAFNSNHQMQLLYQASAMMVESL
NTDRPYKLLIVDSLTSHFRSEYIGRGALAERQQKLARFLAMLHRLANEFDIAVFVTNQVQ
ANGGHILAHSATLRVYLRKGKGGKRIARLIDAPHLPEGEAVFSITEKGIED
>HumRadA16
MATIGRISTGSKSLDKLLGGGIETQAITEVFGEFGSGKTQLAHTLAVMVQLPPEEGGLNG
SAMYIDTENTFRPERIREIAQNRGLDPDEVLKHIAYARAFNSNHQMQLLYQASAMMVELL
NTDRPYKLLIVDSLTSHFRSEYIGRGALAERQQKLARFLRMLHRLANEFDIAVFVTNQVQ
ANGGHILAHSATLRVYLRKGKGGKRIARLIDAPHLPEGEAVFSITEKGIED
>HumRadA18
MATIGRISTGSKSLDKLLGGGIETQAITEVFGEFGSGKTQLAHTLAVMVQLPPEEGGLNG
SAMYIDTENTFRPERIREIAQNRGLDPDEVLDNVAYARAFNSNHQMQLLYQASAMMVESL
NTDRPYKLLIVDSLTSHFRSEYIGRGALAERQQKLARFLAMLHRLANEFDIAVFVTNQVQ
ANGGHILAHSATLRVYLRKGKGGKRIARLIDAPHLPEGEAVFSITEKGIED

>HumRadA20
MATIGRISTGSKSLDKLLGGGIETQAITEVFGEFGSGKTQLAHTLAVMVQLPPEEGGLNG
SAMYIDTENTFRPERIREIAQNRGLDPDEVLDNVAYARAFNSNHQMQLLYQASAMMVESL
NTDRPYKLLIVDSLTSHFRSEYIGRGALAERQQKLARFLRMLHRLANEFDIAVFVTNQVQ
ANGGHILAHSATLRVYLRKGKGGKRIARLIDAPHLPEGEAVFSITEKGIED
>HumRadA22
MATIGRISTGSKSLDKLLGGGIETQAITEVFGEFGSGKTQLAHTLAVMVQLPPEEGGLNG
SAMYIDTENTFRPERLREIAQNRGLDPDEVLDNVAYARAFNSNHQMQLLYQASAMMVESL
NTDRPYKLLIVDSLTSHFRSEYIGRGALAERQQKLARFLRMLHRLANEFDIAVFVTNQVQ
ANGGHILAHSATLRVYLRKGKGGKRIARLIDAPHLPEGEAVFSITEKGIED
>HumRadA33
MATIGRISTGSKSLDKLLGGGIETQAITEVFGEFGSGKTQLAHTLAVMVQLPPEEGGLNG
KAMYIDTEGTFRPERLLEIAQNRGLSGSDVLDNVAYARAFNSNHQMQLLYQASAMMVESL
NTDRPYKLLIVDSLTSHFRSEYIGRGALAERQQKLARFLRMLHRLANEFDIAVFVTNQVQ
ANGGHILAHSATLRVYLRKGKGGKRIARLIDAPHLPEGEAVFSITEKGIED
>HumRadA22F
MATIGRISTGSKSLDKLLGGGIETQAITEVFGEFGSGKTQLAHTLAVMVQLPPEEGGLNG
SAMYIDTENTFRPERLREIAQNRGLDPDEVLDNVAYARAFNSNHQMLLVQQAEDMIKELL
NTDRPVKLLIVDSLTSHFRSEYIGRGALAERQQKLAKHLADLHRLANLYDIAVFVTNQVQ
ANGGHILAHSATLRVYLRKGKGGKRIARLIDAPHLPEGEAVFSITEKGIED
>HumRadA26F
MATIGRISTGSKSLDKLLGGGIETQAITEVFGEFGSGKTQLAHTLAVMVQLPPEEGGLNG
KAMYIDTENTFRPERLLEIAQNRGLDPDEVLDNVAYARAFNSNHQMLLVQQASDMMKELL
NTDRPVKLLIVDSLTSHFRSEYIGRGALAERQQKLAKHLADLHRLANLYDIAVFVTNQVQ
ANGGHILAHSATLRVYLRKGKGGKRIARLIDAPHLPEGEAVFSITEKGIED
>HumRadA28F
MATIGRISTGSKSLDKLLGGGIETQAITEVFGEFGSGKTQLAHTLAVMVQLPPEEGGLNG
KAMYIDTENTFRPERLREIAQNRGLDPDEVLDNVAYARAFNSNHQMQLLYQASDMMVESL
NTDRPYKLLIVDSLTSHFRSEYIGRGALAERQQKLARFLRMLHRLANEFDIAVFVTNQVQ
ANGGHILAHSATLRVYLRKGKGGKRIARLIDAPHLPEGEAVFSITEKGIED
>HumRadA33F
MATIGRISTGSKSLDKLLGGGIETQAITEVFGEFGSGKTQLAHTLAVMVQLPPEEGGLNG
KAMYIDTEGTFRPERLLEIAQNRGLSGSDVLDNVAYARAFNSNHQMLLVQQASDMMVELL
NTDRPYKLLIVDSLTSHFRSEYIGRGALAERQQKLAKHLADLHRLANLYDIAVFVTNQVQ
ANGGHILAHSATLRVYLRKGKGGKRIARLIDAPHLPEGEAVFSITEKGIED
>flChimRAD51
MAMQMQLEANADTSVEEESFGPQPISRLEQCGINANDVKKLEEAGFHTVEAVAYAPKKEL
INIKGISEAKADKILAEAAKLVPMGFTTATEFHQRRATIGRISTGSKSLDKLLGGGIETQ
AITEVFGEFGSGKTQLAHTLAVMVQLPPEEGGLNGKAMYIDTEGTFRPERLLAVAERYGL
SGSDVLDNVAYARAFNTDHQTQLLYQASAMMVESRYALLIVDSATALYRTDYSGRGELSA
RQMHLARFLRMLLRLADEFGVAVFVTNQVQARPDAFFGDPTRPIGGHILAHSATLRVYLR
KGKGGKRIARLIDAPHLPEGEAVFSITEKGIED
>ChimRAD51
MATIGRISTGSKSLDKLLGGGIETQAITEVFGEFGSGKTQLAHTLAVMVQLPPEEGGLNG
KAMYIDTEGTFRPERLLAVAERYGLSGSDVLDNVAYARAFNTDHQTQLLYQASAMMVESR
YALLIVDSATALYRTDYSGRGELSARQMHLARFLRMLLRLADEFGVAVFVTNQVQARPDA
FFGDPTRPIGGHILAHSATLRVYLRKGKGGKRIARLIDAPHLPEGEAVFSITEKGIED

```
>flHumRadA22
MAGEEVKEIDEFEELGFEPATEETPKKKKKEKIIRSIEDLPGVGPATAEKLREAGYDTLE
AIAVASPIELKEVAGISEGTALKIIQAARKAANLGTFMRADEYLKKRATIGRISTGSKSL
DKLLGGGIETQAITEVFGEFGSGKTQLAHTLAVMVQLPPEEGGLNGSAMYIDTENTFRPE
RLREIAQNRGLDPDEVLDNVAYARAFNSNHQMQLLYQASAMMVESLNTDRPYKLLIVDSL
TSHFRSEYIGRGALAERQQKLARFLRMLHRLANEFDIAVFVTNQVQARPDAFFGDPTRPI
GGHILAHSATLRVYLRKGKGGKRIARLIDAPHLPEGEAVFSITEKGIED
>HsRAD51 (wild type human RAD51)
MAMQMQLEANADTSVEEESFGPQPISRLEQCGINANDVKKLEEAGFHTVEAVAYAPKKEL
INIKGISEAKADKILAEAAKLVPMGFTTATEFHQRRSEIIQITTGSKELDKLLQGGIETG
SITEMFGEFRTGKTQICHTLAVTCQLPIDRGGGEGKAMYIDTEGTFRPERLLAVAERYGL
SGSDVLDNVAYARAFNTDHQTQLLYQASAMMVESRYALLIVDSATALYRTDYSGRGELSA
RQMHLARFLRMLLRLADEFGVAVVITNQVVAQVDGAAMFAADPKKPIGGNIIAHASTTRL
YLRKGRGETRICKIYDSPCLPEAEAMFAINADGVGDAKD
>PfRadA (wild type Pyrococcus furiosus RadA)
MAGEEVKEIDEFEELGFEPATEETPKKKKKEKIIRSIEDLPGVGPATAEKLREAGYDTLE
AIAVASPIELKEVAGISEGTALKIIQAARKAANLGTFMRADEYLKKRATIGRISTGSKSL
DKLLGGGIETQAITEVFGEFGSGKTQLAHTLAVMVQLPPEEGGLNGSVIWIDTENTFRPE
RIREIAQNRGLDPDEVLKHIYVARAFNSNHQMLLVQQAEDKIKELLNTDRPVKLLIVDSL
TSHFRSEYIGRGALAERQQKLAKHLADLHRLANLYDIAVFVTNQVQARPDAFFGDPTRPI
GGHILAHSATLRVYLRKGKGGKRIARLIDAPHLPEGEAVFSITEKGIED
```
